# Magnetoelectric Nanotherapy Achieves Complete Tumor Ablation and Prolonged Survival in Pancreatic Cancer Murine Models

**DOI:** 10.1002/advs.202517228

**Published:** 2025-11-03

**Authors:** John Michael Bryant, Max Shotbolt, Emmanuel Stimphil, Victoria Andre, Elric Zhang, Veronica Estrella, Kazim Husain, Joseph Weygand, Doug Marchion, Alex Sebastian Lopez, Dominique Abrahams, Shawnus Chen, Mostafa Abdel‐Mottaleb, Skye Conlan, Ibrahim Oraiqat, Vaseem Khatri, Jose Alejandro Guevara, Shari Pilon‐Thomas, Gage Redler, Kujtim Latifi, Natarajan Raghunand, Kosj Yamoah, Sarah Hoffe, James Costello, Jessica M. Frakes, Ping Liang, Robert A. Gatenby, Mokenge Malafa, Sakhrat Khizroev

**Affiliations:** ^1^ Department of Radiation Oncology University of Miami Miller School of Medicine Miami FL 33136 USA; ^2^ Department of Radiation Oncology H. Lee Moffitt Cancer Center and Research Institute Tampa FL 33612 USA; ^3^ College of Engineering University of Miami Coral Gables FL 33146 USA; ^4^ Cancer Physiology/Clinical Science Laboratory H. Lee Moffitt Cancer Center and Research Institute Tampa FL 33612 USA; ^5^ The Institute of Robotics and Intelligent Systems Swiss Federal Institute of Technology (ETH) Zurich Zurich 8092 Switzerland; ^6^ Department of Gastrointestinal Oncology H. Lee Moffitt Cancer Center and Research Institute Tampa FL 33612 USA; ^7^ Department of Radiation Oncology and Applied Sciences Geisel School of Medicine Dartmouth College Hanover NH 03755 USA; ^8^ Tissue Core H. Lee Moffitt Cancer Center and Research Institute Tampa FL 33612 USA; ^9^ Department of Comparative Medicine University of South Florida Tampa FL 33612 USA; ^10^ Department of Immuno‐Oncology H. Lee Moffitt Cancer Center and Research Institute Tampa FL 33612 USA; ^11^ Department of Diagnostic Imaging and Interventional Radiology H. Lee Moffitt Cancer Center and Research Institute Tampa FL 33612 USA; ^12^ Cellular Nanomed Inc. Irvine CA 92617 USA; ^13^ Department of Biochemistry and Molecular Biology Miller School of Medicine University of Miami Coral Gables FL 33136 USA

**Keywords:** magnetoelectric nanoparticles, pancreatic cancer, theranostics

## Abstract

Magnetoelectric nanoparticles (MENPs), when activated by a magnetic field, are shown to provide a minimally invasive, drug‐free, theranostic approach to pancreatic ductal adenocarcinoma (PDAC) treatment. The magnetoelectric effect allows intravenously administered MENPs to be magnetically guided to PDAC tumors and remotely activated with a 7T‐MRI field to induce targeted, electrode‐free tumor ablation with real‐time imaging feedback. A single MENP treatment achieved a threefold median reduction in tumor volume and complete tumor responses in 33.3% of mice at 300 and 600 µg doses (*N* = 17) and significantly longer mean overall survival as compared to the control cohorts (54.1 vs 28.8 days, χ^2^ = 40.14, *p* = 0.045), without evident toxicity in any imaged organ. In contrast, mice receiving subtherapeutic doses, non‐activated MENPs, or saline controls showed no significant response. MRI T_2_* relaxation time decreases closely correlated with tumor reduction (ρ = −0.73, *p* < 0.001), supporting MENPs as both a therapeutic and imaging biomarker. Mechanistically, MENPs preferentially target cancer cells via magnetic‐field‐driven electrostatic interactions specific to tumor cell membranes, in agreement with multiphysics numerical simulations. Flow cytometry confirmed that MENP activation primarily induces apoptosis, with minimal necrosis, and time‐course studies showed a progressive apoptotic response over 3‐hour post‐treatment. The findings establish MENPs as a versatile, image‐guided, theranostic platform with translational promise for minimally invasive oncology.

## Graphical abstract

Magnetoelectric nanoparticles (MENPs) serve as externally controlled, MRI‐activated theranostic agents for targeted cancer therapy by inducing electric field‐based ablation in solid tumors. MENPs enable simultaneous precise tumor ablation and MRI signal modulation, allowing real‐time treatment monitoring and prediction of therapeutic outcomes with no discernable toxicity in preclinical models.

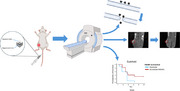

## 1 Introduction

Pancreatic ductal adenocarcinoma (PDAC) is an aggressive malignancy characterized by late presentation, resistance to standard therapies, and a uniquely challenging microenvironment marked by a dense stromal matrix and profound immunosuppression.^[^ ^1^ ^]^ Despite recent advances in immunotherapy and targeted approaches, the median survival remains dismal: most patients present with unresectable disease, and 5‐year survival rates are below 10% in most cohorts. Epidemiological projections indicate that PDAC is poised to become the second leading cause of cancer‐related death in the United States by 2030,^[^ ^1^ ^]^ underscoring the urgent clinical need for effective new treatment modalities.

Among emerging local therapies, electric field‐based therapies such as tumor treating fields (TTFs) and irreversible electroporation (IRE) have garnered significant attention for their ability to ablate tumors while preserving critical anatomical structures.^[^ ^2^, ^3^ ^]^ IRE induces cell death via high‐voltage electric pulses that permeabilize cellular membranes, leading to tumor ablation. Early trials in PDAC have demonstrated promising local control and the potential of converting an immunosuppressive tumor microenvironment (TME) into an immunopermissive one.^[^ ^4^, ^5^, ^6^ ^]^ However, despite the efficacy and potential of IRE in oncology, several limitations have restricted its clinical adoption, including the invasive nature of electrode placement, the need for general anesthesia, and the risk of off‐target tissue damage. TTFs are shown to have a number of concurrent mechanisms for treating various cancers, particularly through disrupting mitotic spindles. TTF therapy is limited by the indefinite duration of treatment, the need to wear the device for most of the day (often up to 20 h), and the potential for skin irritation, among other challenges.

To overcome these barriers, we engineered a new geometry of magnetoelectric nanoparticles to function as a minimally invasive, drug‐free theranostic platform.^[^ ^7^ ^]^ MENPs utilize the magnetoelectric effect – allowing conversion of externally applied magnetic fields into highly localized electric fields – at the tumor site without physical electrodes or systemic toxicity.^[^ ^8^, ^9^, ^10^, ^11^, ^12^, ^13^, ^14^, ^15^, ^16^, ^17^ ^]^ After intravenous administration, MENPs can be magnetically guided to the tumor microenvironment.^[^ ^18^ ^]^ We show that subsequent activation by the magnetic field of a clinical MRI induces local electric fields sufficient to drive tumor ablation,^[^ ^19^, ^20^ ^]^ while providing simultaneous MRI‐based imaging feedback.^[^ ^21^, ^22^, ^23^ ^]^ This dual functionality not only addresses the delivery and precision limitations of conventional IRE but also enables real‐time noninvasive monitoring of therapeutic response, an unmet need in the clinical management of PDAC.^[^ ^24^, ^25^, ^26^, ^27^ ^]^

Mechanistically, MENP targeting exploits tumor‐selective dielectric properties, enabling preferential membrane interaction with cancer cells over healthy tissue in response to weak guiding fields. As demonstrated in our prior theoretical and comprehensive simulation work, and now in murine models, the force interactions between MENPs and cell membranes—including magnetic, van der Waals, and electrostatic effects—facilitate highly specific cancer cell targeting at a single‐cell level.^[^ ^28^, ^29^, ^30^, ^31^ ^]^ Upon MRI activation, MENPs generate localized fields that trigger irreversible membrane permeabilization and cell death, predominantly via apoptotic pathways, verified both in vitro and in vivo.^[^ ^32^, ^33^ ^]^ When combined with quantitative MRI T_2_* relaxometry, MENPs offer a fully integrated theranostic approach, bridging targeted therapy and imaging in a single platform.^[^ ^34^, ^35^, ^36^ ^]^

To date, the most widely used MENP system has a core‐shell structure, e.g., CoFe_2_O_4_@BaTiO_3_.^[^ ^37^ ^]^ Because of strain propagation at the lattice‐matched interface between the magnetostrictive core and the piezoelectric shell, this nanocomposite system exhibits a relatively high ME coefficient, in turn enabling efficient local conversion between magnetic and electric fields.^[^ ^38^ ^]^

Here, we present the first in vivo study in a murine model to evaluate the efficacy, selectivity, and safety of systemically delivered, MRI‐activated MENPs for PDAC. We demonstrate robust, dose‐dependent tumor ablation, durable complete responses, and a strong correlation between MRI signal changes and therapeutic outcomes—alongside no measurable toxicity.

## 2 Results

### 2.1 MENP‐Driven Reactive Oxygen Species Generation and Induction of Apoptotic Cell Death In Vitro

**Figure 1:**
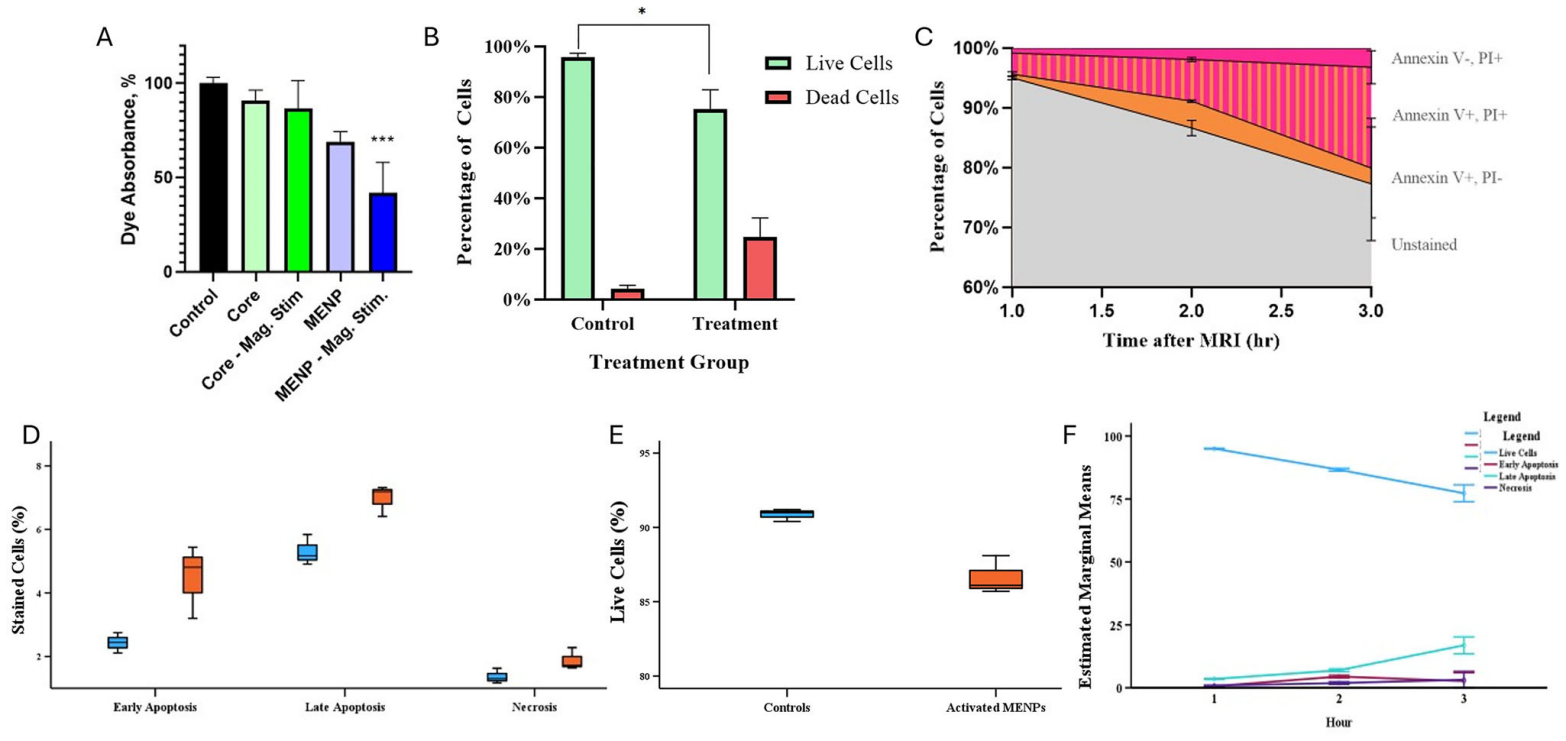
MENP characterization and in vitro cell‐kill mechanism. A) Trypan Blue dye test optimization of the ME effect of MENPs. Standard MENPs had no significant effect on the dye solution on their own, but upon stimulation with an AC magnetic field, the dye absorption was reduced to 37.3% of the original absorption, as demonstrated by a Friedman test and Dunn's multiple comparison test (*p* < 0.01). MENP cores (i.e., naked ferrimagnetic cores) were tested as a control and validation of the test and did not have any effect on the dye absorption regardless of AC stimulation. B) Cell death percentage as measured by Trypan Blue assay. MENP samples demonstrated a significant increase in dead cells (*p* =.0097). C) Annexin V and PI uptake over time after treatment with MENPs and MRI. A steady decrease in unstained cells demonstrated cell death over 3 h after treatment. An increase in Annexin V–positive and PI‐positive cells over time is indicative of apoptotic cells with permeable membranes. D) Percentage of cells killed through early apoptosis, late apoptosis, and necrosis 2‐h after treatment. Student's t‐test demonstrated that activated MENPs predominantly induce apoptosis (both early and late) with significant increases compared to controls (*p* < 0.05). Necrosis remains minimal, and there was not a significant difference between MENPs and controls, emphasizing the apoptotic nature of the MENP‐induced cell‐kill mechanism. E) Percentage of live KPC961 cells after treatment with activated MENPs compared to controls. Student's t‐test demonstrated that the activated MENPs significantly reduced cell viability (*p* < 0.01), demonstrating their potent cytotoxic effect upon AC magnetic field stimulation. Controls maintained high viability, further confirming the specificity of the MENP‐mediated mechanism. F) MRI Activated MENP early cell‐kill characterization. Time‐dependent progression of cell death pathways in KPC961 cells treated with activated MENPs. Early apoptosis predominates within the first hour, transitioning to late apoptosis over time, while necrosis remains minimal throughout the observation period. This progression highlights the controlled and predictable apoptotic mechanism of MENP activation. Error bars represent the mean ± 1 standard error. Significant associations are indicated as follows: ^*^ for *p* < 0.05, ^**^ for *p* < 0.01, and ^***^ for *p* < 0.001. Abbreviations: AC: alternating current; KPC961: Kras mutant pancreatic cancer cell line; ME: magnetoelectric; MENP: magnetoelectric nanoparticle; MRI: magnetic resonance imaging.

To assess whether MENPs could generate electric‐field effects upon magnetic activation, we first evaluated their ability to induce dye degradation as a surrogate for reactive oxygen species (ROS) generation. Trypan blue solutions incubated with two variants of MENPs were subjected to sonication alone or concomitant 1kHz, 0.025T alternating current (AC) magnetic field stimulation. Only the magnetically stimulated, high ME coefficient group exhibited a significant decrease in absorbance (to 37.3% of baseline, *p* = 0.007), indicating ROS‐driven dye degradation, while MENPs without field activation had no effect, as shown in **Figure** **1**A. This demonstrates that MENPs efficiently convert applied magnetics energy into localized electric fields, driving ROS generation only under field stimulation.

Next, we examined whether MRI‐activated MENPs induce cancer cell death in vitro. Murine KPC‐961 pancreatic cancer cells were incubated with and without MENPs and exposed to 7T MRI magnetic field activation. Flow cytometry using Annexin V/PI staining revealed a significant reduction in live cells in the MRI‐activated MENP group compared to controls (*p* = 0.006; Figure 1B). This cytotoxicity was characterized by a marked increase in early (*p* = 0.041) and late (*p* = 0.014) apoptotic cells, with negligible necrosis observed between groups (Figure 1C–F), indicating apoptosis as the dominant cell death mechanism.

A time‐course analysis further demonstrated progressive, predominantly apoptotic, cell death in MENP‐treated samples following MRI activation. At 1 h, most dying cells were in late apoptosis; at 2 and 3 h, there was a transition from early to late apoptotic states, while necrosis remained minimal at all time points (Figure 1C). These findings indicate that MRI‐activated MENPs selectively induce cancer cell apoptosis via magnetoelectric field‐driven mechanisms, providing evidence of both specificity and mechanism for subsequent in vivo studies.

### 2.2 Preliminary Tumor Ablation and T_2_* Signal Modulation In Vivo Investigation

**Figure 2:**
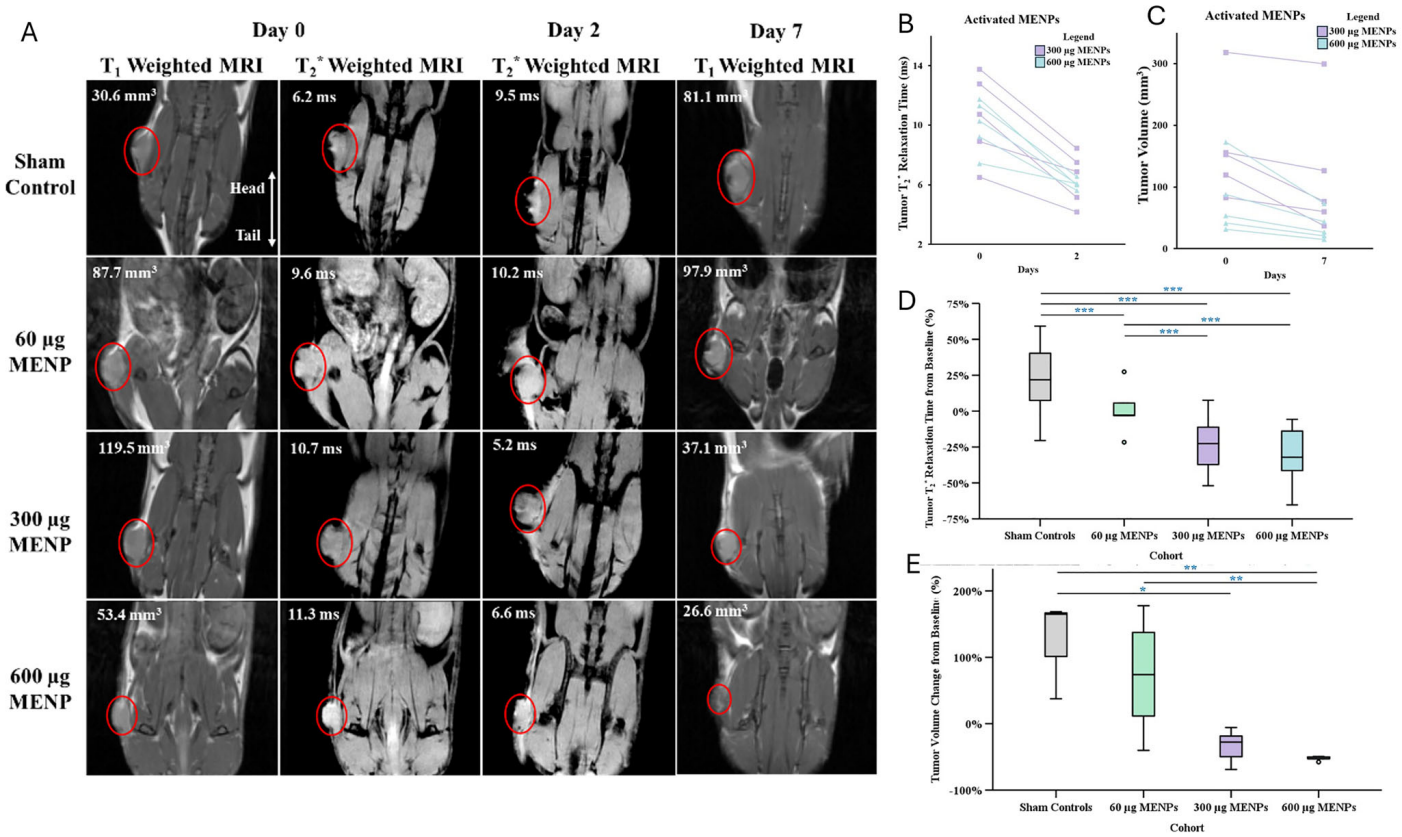
Tumor volume and relaxation time outcomes. A) Representative MRI images across cohorts at Days 0, 2, and 7. Images display T_1_‐weighted and T_2_^*^‐weighted sequences. Both T_1_ and T_2_^*^ images were acquired using gradient echo sequences; however, relaxation time measurements shown were performed on mapping sequences (not shown). Tumors (circled in red) treated with 300 and 600 µg of MRI‐activated MENPs showed intratumoral darkening contrast and were visibly decreased on day 7. Significant associations are indicated as follows: ^*^ for *p* < 0.05, ^**^ for *p* < 0.01, and ^***^ for *p* < 0.001. Blue asterisks indicate unadjusted significance (LSD and Dunn's), and green asterisks indicate adjusted significance (Bonferroni). Abbreviations: KW: Kruskal‐Wallis; MRI: magnetic resonance imaging; MENP: magnetoelectric nanoparticle. B) Tumor T_2_^*^ relaxation times for activated MENP cohorts (300 µg and 600 µg) on Days 0 and 2. The T_2_^*^ relaxation times are reduced in all tumors with activated MENPs. C) Tumor volume measurements of activated MENP cohorts (300 and 600 µg) on days 0 and 7. Both cohorts demonstrated significant tumor volume reduction over time, with greater reductions observed in the 600 µg MENP group. D) Percent change in tumor T_2_^*^ relaxation times between days 0 and 2. One‐way ANOVA (F = 29.133, df = 3, *p* = <0.001) demonstrated highly significant differences between all the cohorts on Bonferroni correction, except for between the 300 and 600 µg MENP cohorts. E) Bar chart of percent change in tumor volume by day 7. KW analysis for tumor volume changes from baseline across cohorts revealed a significant difference (H = 11.95, df = 3, *p* = 0.008). Sham controls exhibited minimal tumor volume change, whereas MENP‐treated cohorts displayed significant tumor volume reductions in a dose‐dependent manner. The 600 µg MENP cohort demonstrated significant reductions as compared to both the sham controls and the 60 µg MENP cohort (adjusted *p* < 0.05).

To evaluate whether systemically administered MENPs coupled with MRI activation achieve targeted tumor ablation and modulate MRI signals in vivo, we first conducted a small‐scale randomized study in a murine PDAC model. Eighteen C57BL/6 mice bearing KPC‐961 flank tumors (median baseline volume 87.9 mm^3^, IQR 44.4–144.1 mm^3^) were randomized to receive intravenous injections of saline (control, *N* = 3), high ME MENPs doses (60, 300, and 600 µg, *N* = 5 each), followed by magnetic targeting with a 0.15 T neodymium magnet applied over the tumor. MR imaging (T_2_*, T_2_, and T_1_ mapping) was conducted pre‐treatment (day 0), post‐injection and activation (day 2), and at follow‐up (day 7) (**Figure** **2**A). Initial weights and tumor volumes are shown in Figure S3 (Supporting Information).

MRI images illustrate tumor volume and T_2_* changes across dose groups and the sham control group (Figure 2A). MRI T_2_* relaxation times within tumors decreased significantly in the 300 and 600 µg cohorts, indicating MENP accumulation and activation‐induced intratumoral changes. Reductions in T_2_* relaxation times were marked relative to controls (300 µg: r = 0.657, *p* = 0.032, CI: [‐24.9, ‐51.2] ms; 600 µg: r = 0.681, *p* = 0.023, CI: [−23.4, −52.3] ms), whereas the 60 µg group showed no significant modulation (Figure 2D). Representative MRI images illustrate tumor volume and T_2_* changes across dose groups (Figure 2A). Absolute volume changes are shown in Figure S4 (Supporting Information).

Concurrently, tumor volume analysis revealed significant dose‐dependent treatment responses. Mice receiving 300 and 600 µg MENP doses exhibited consistent tumor shrinkage by day 7, with median reductions of approximately threefold relative to baseline(300 µg: CI: [−20.7, −51.7] mm^3^; 600 µg: CI: [−22.6, −55.6] mm^3^), whereas low‐dose (60 µg) and saline controls showed no significant tumor regression (Figure 2B–E). The 600 µg group demonstrated a statistically significant tumor volume reduction compared to controls (r = 0.673, *p* = 0.026) and the 60 µg group (r = 0.642, *p* = 0.039), though the difference between 300 and 600 µg was not significant (Figure 2D). No significant difference in baseline tumor size or mouse weight was observed between groups (Figure S3E, Supporting Information).

Sex‐based analyses found no significant differences in tumor volume or T_2_* signal modulation between male and female mice (*p* > 0.4 for both; Figure S5, Supporting Information), confirming treatment effects were independent of sex.

These data points confirm that MRI‐activated MENPs induce robust, dose‐dependent tumor ablation correlating with quantifiable T_2_* signal changes, illustrating their theranostic capability for simultaneous treatment and imaging, example images and contouring shown in Figures S11 and S12 (Supporting Information).

### 2.3 Validation of MENP‐Induced Tumor Ablation and Toxicity Profile with Longitudinal In Vivo Assessment

To confirm the reproducibility, durability, and safety of MENP‐mediated tumor ablation observed in the pilot cohort described above, we performed a follow‐up, longitudinal study and concurrent organ toxicity evaluation. Twenty‐seven female C57BL/6 mice bearing right flank PDAC tumors (KPC‐961) were randomized to receive saline (control, *N* = 5), 300 µg MENPs (*N* = 6), 600 µg MENPs (*N* = 6), non‐activated MENPs (300 µg, *N* = 5), or 300 µg MENPs with reduced ME coefficient (*N* = 5), with all procedures standardized as in the initial experiment. Baseline measurements are shown in Figure S6 (Supporting Information).

**Figure 3:**
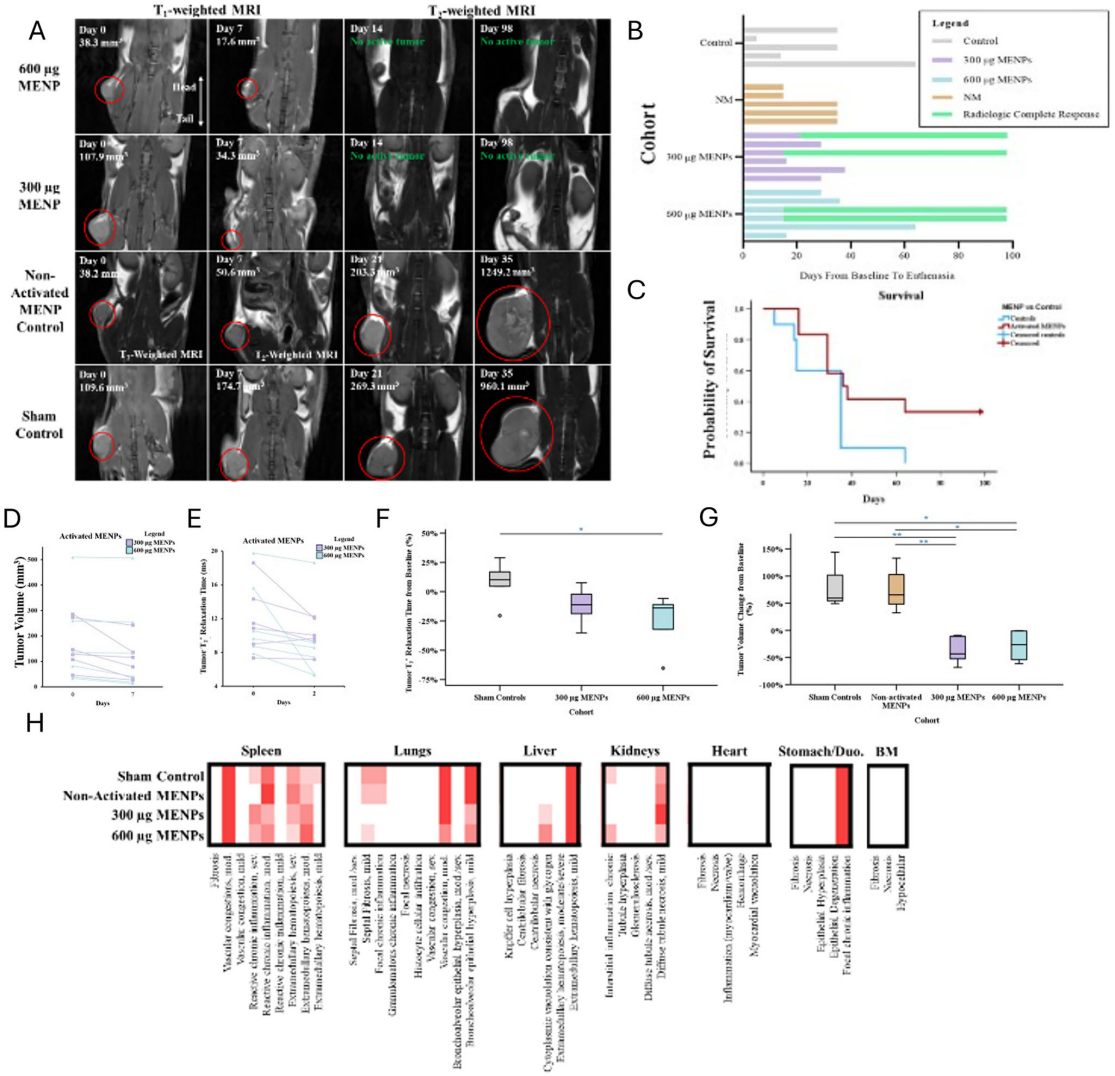
Tumor control, survival, and treatment‐associated toxicity outcomes in a confirmatory MENP study. A) Representative MRI images of tumor volumes for sham, non‐activated MENP, 300 µg MENP, and 600 µg MENP cohorts. Tumor shrinkage and CR are evident in the 300 and 600 µg cohorts by day 14, which remain stable over the remaining 84 days. All mice within the control cohorts experienced continued tumor growth until reaching predetermined tumor euthanasia endpoints. B) Time‐to‐event waterfall plot of confirmatory study. Two mice within the 300 and 600 µg cohorts (ie, 4 mice in total) achieved a CR (as determined via exam and MRI). After achieving a CR, none of the mice experienced any evidence of disease recurrence. All other mice had eventual tumor growth that met predetermined euthanasia endpoints. C) Kaplan‐Meier survival analysis of activated MENP vs. control cohorts. Mice treated with activated MENPs (300 and 600 µg) demonstrated a significant improvement in survival compared to the control group, with a mean survival time of 54.1 days (95% CI: 35.3 to 72.8) vs 28.8 days (95% CI: 18.3–39.3, χ^2^ = 40.14, *p* = 0.045). D) Absolute tumor volume measurements for activated MENP cohorts (300 and 600 µg) on Days 0 and 7. No mice in activated MENP cohorts experienced tumor growth on day 7. E) Absolute tumor T_2_^*^ relaxation times for activated MENPs (300 and 600 µg) between Days 0 and 2. Significant reductions in 91.7% of intratumoral T_2_^*^ relaxation times were observed, consistent with effective MENP‐mediated tumor targeting. F) Bar chart of percent change in tumor T_2_^*^ relaxation times between Days 0 and 7 across all cohorts. One‐way ANOVA (F = 3.939, df = 3, *p* = 0.044) revealed a significant difference in tumor T_2_^*^ relaxation times as before; however, only the 600 µg MENP cohort of activated MENPs demonstrated significant reductions in T_2_^*^ values as compared to sham controls on adjusted significance (*p* = 0.043). G) Bar chart of percent change in tumor volume between day 0 and day 7 across all cohorts. KW analysis for tumor volume changes from baseline across cohorts revealed a significant difference (H = 14.88, df = 3, *p* = 0.002). Sham controls and non‐activated MENP cohorts did not exhibit any tumor control, while activated MENPs (300 and 600 µg) demonstrated dose‐dependent tumor regression (*p* < 0.01 for adjusted and unadjusted comparisons). H) Heat maps summarizing histopathological findings in organs, including the spleen, lungs, liver, kidneys, heart, stomach/duodenum, and bone marrow. No significant differences were observed in the activated MENP cohorts as compared to sham and non‐activated MENP cohorts, confirming the safety of the treatment. Significant associations are indicated as follows: ^*^ for *p* < 0.05, ^**^ for *p* < 0.01, and ^***^ for *p* < 0.001. Blue asterisks indicate unadjusted significance (LSD and Dunn's), and green asterisks indicate adjusted significance (Bonferroni). Abbreviations: BM: bone marrow; CR: complete response; KW: Kruskal‐Wallis; MRI: magnetic resonance imaging; MENP: magnetoelectric nanoparticle; RARE: rapid acquisition with relaxation enhancement; sev.: severe; mod.: moderate.

MRI images illustrate tumor volume and T_2_* changes across two main dose groups (300 and 600 µg) and two control groups (sham and non‐activated MENPs) (**Figure** **3**A).

Baseline characteristics—including tumor volume, T_2_* relaxation times, and mouse weights—were comparable across all groups (Table S2, Supporting Information). Both 300 and 600 µg MENP treatment groups exhibited robust and consistent tumor shrinkage by day 7 (median reductions ≈30–35% from baseline, Figure 3C,E), with effect sizes similar or greater than those observed in the initial cohort and achieving statistical significance versus controls (300 µg: r = 0.859, *p* = 0.026; 600 µg: r = 0.759, *p* = 0.071; Figure 3D; Figure S7B, Supporting Information). Non‐activated MENP and control groups showed continued tumor growth. Importantly, T_2_* relaxation times decreased markedly in the majority of MENP‐activated mice (91.7% [11/12]; Figure 3A,B,D), again paralleling treatment response. Absolute measurement changes are shown in Figure S7 (Supporting Information).

Longitudinal monitoring over 14 weeks revealed that all mice in both the control and non‐activated MENP cohorts exhibited progressive tumor growth at all time points. In contrast, 33.3% (4/12) of MENP‐activated mice achieved durable complete tumor responses—confirmed by serial MRI and physical examination—with sustained tumor absence up to study endpoint (Figure 3H,I). Overall survival was significantly extended in MENP‐treated mice versus controls (mean 54.1 vs. 28.8 days, χ^2^ = 40.14, *p* = 0.045; Figure 3H), driven primarily by the rate of complete responses. Absolute volume changes over time for each mouse are shown in Figure S8 (Supporting Information).

Serial health and weight assessments indicated no significant differences in adverse effects, behavior, or weight change between experimental cohorts at any time point. Comprehensive histopathologic evaluation of heart, lungs, liver, spleen, kidneys, stomach, duodenum, and bone marrow at study endpoint revealed no statistically significant or clinically relevant organ toxicity in any MENP‐treated group compared to controls (Figure 3J). Weight changes for mice are given in Figure S9 (Supporting Information), and data are summarized in Tables S1 and S2 (Supporting Information).

These findings validate the reproducibility, durability, and favorable safety profile of systemically delivered, MRI‐activated MENPs for in vivo tumor ablation. The absence of discernible toxicities, durable complete responses, and significant survival benefit supports the translational potential of MENPs as a minimally invasive, theranostic cancer therapy.

### 2.4 Follow‐Up MENP Dose per Tumor Volume and Theranostic Potential In Vivo

Recognizing that baseline tumor volume could impact therapeutic efficacy, we investigated whether normalizing MENP dose to tumor size better predicts treatment outcomes. We combined datasets from the pilot and validation cohorts after confirming comparable baseline characteristics and experimental conditions. A two‐way ANOVA revealed a significant interaction between dose and baseline tumor volume on tumor response (*p* = 0.020), suggesting that absolute dose alone is insufficient and dose relative to tumor volume is a critical determinant of efficacy.

**Figure 4:**
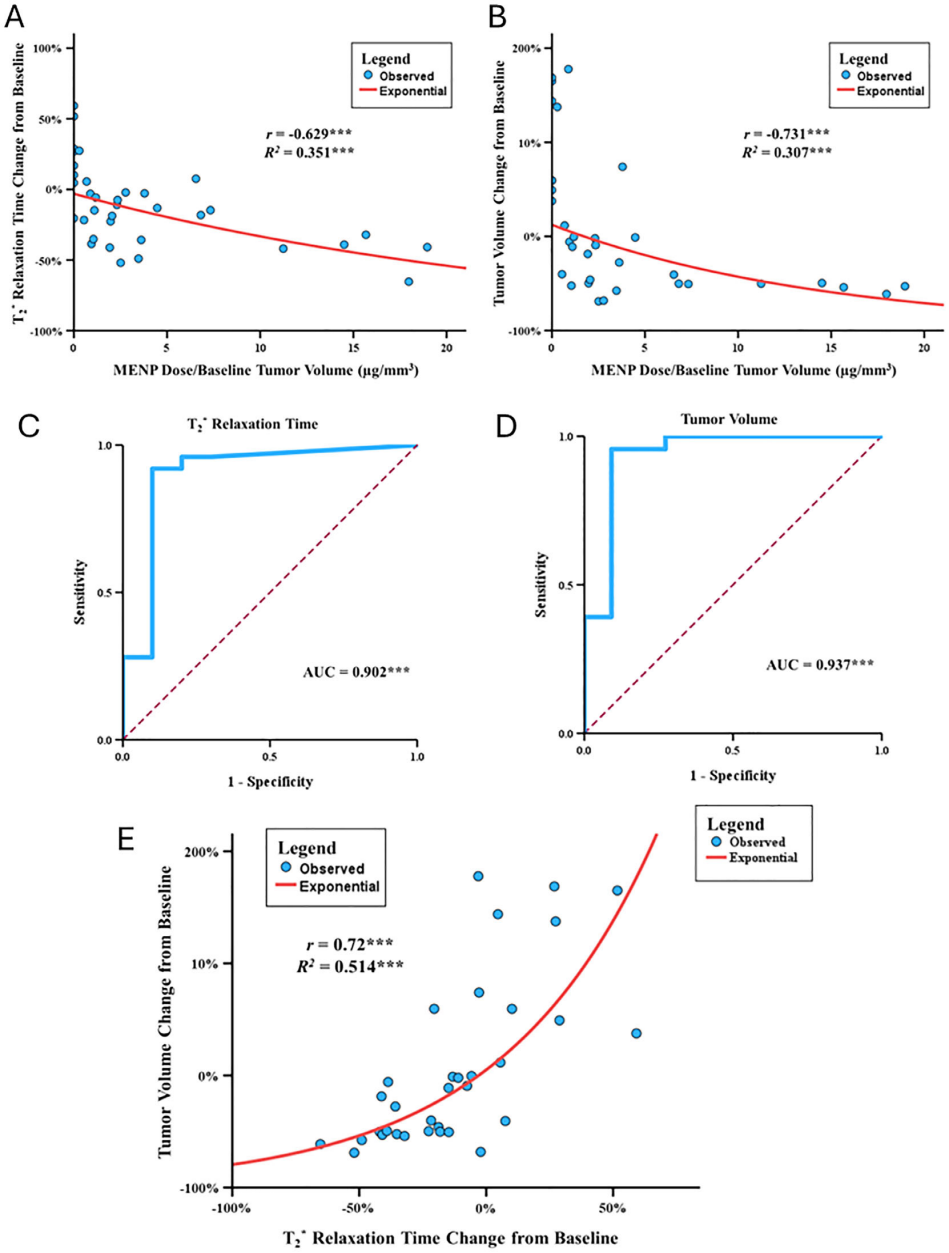
Dose per tumor volume normalization and its correlation with therapeutic outcomes. A) Relationship between MENP dose per baseline tumor volume (µg/mm^3^) and T_2_^*^ relaxation time change from baseline (%). A similar significant exponential decay was observed, with higher normalized doses correlating with greater decreases in T_2_^*^ relaxation times (*r* = −0.629, *R^2^* = 0.351, *p* < 0.001). B) Relationship between MENP dose per baseline tumor volume (µg/mm^3^) and tumor volume change from baseline. A significant exponential decay was observed, with higher normalized doses correlating with greater reductions in tumor size (*r* = −0.731, *R^2^* = 0.307, *p* < 0.001). C) ROC AUC analysis for T_2_* relaxation time reduction as a function of dose per tumor volume. A strong predictive capacity was similarly observed (AUC = 0.902, 95% CI: 0.762–1.000, *p* < 0.001), with the Youden's J statistic identifying a threshold dose per tumor volume of 0.784 µg mm^−^^3^. D) ROC AUC analysis for tumor volume reduction as a function of dose per tumor volume. The analysis revealed a strong predictive capacity (AUC = 0.937, 95% CI: 0.829–1.000, *p* < 0.001), with Youden's J statistic identifying a threshold dose per tumor volume of 0.931 µg mm^−^^3^. E) Relationship between T_2_* relaxation time change from baseline (%) and tumor volume change from baseline. A significant positive correlation was found (*r* = 0.72, *R^2^* = 0.514, *p* < 0.001), following an exponential trend. Larger reductions in T_2_* relaxation time were strongly associated with subsequent tumor shrinkage, supporting the role of T_2_* as an early indicator of MENP therapeutic efficacy. Significant associations are indicated as follows: ^*^ for *p* < 0.05, ^**^ for *p* < 0.01, and ^***^ for *p* < 0.001.

Using dose normalized by baseline tumor volume (µg MENPs/mm^3^ tumor), Spearman correlation analysis demonstrated strong negative correlations with tumor volume reduction (ρ = −0.73, *p* < 0.001) and T_2_* relaxation time decrease (ρ = −0.63, *P* < 0.001), indicating higher normalized doses yield greater tumor shrinkage and MRI signal modulation (**Figure** **4**A–B). The relationship between normalized dose and tumor volume response followed a significant exponential decay model (R^2^ = 0.307, *P* < 0.001), and a similar model described the T_2_* change (R^2^ = 0.351, *P* < 0.001).

To identify thresholds predictive of therapeutic benefit, we performed receiver operating characteristic (ROC) curve analyses. The area under the curve (AUC) was 0.937 (*P* < 0.001) for tumor volume reduction and 0.902 (*p* < 0.001) for T_2_* relaxation time change, demonstrating high predictive power of dose per tumor volume (Figure 4C,D). Using Youden's J statistic, an optimal dose threshold of 0.931 µg mm^−^^3^ was identified for tumor volume reduction and 0.784 µg mm^−^^3^ for significant T_2_* changes.

Tumors receiving doses above these cutoffs experienced mean tumor volume reductions of −32.8% (95% CI: −46.9%–−18.7%) and T_2_* reduction of −25.9% (95% CI: −34.0%–−17.8%). Notably, changes in T_2_* relaxation times closely correlated with subsequent tumor volume reduction (ρ = 0.72, *p* < 0.001; Figure 4E), supporting T_2_* as a potential early imaging biomarker of MENP therapeutic efficacy.

## 3 Discussion

This study provides the first in vivo validation of a class of magnetoelectric nanoparticles as a wireless field‐activated, drug‐free, theranostic platform for targeted tumor ablation with real‐time imaging feedback. MENPs uniquely enable the non‐contact induction and reciprocal detection of localized electric fields within biological systems, overcoming major limitations of conventional approaches that rely on implanted electrodes or restricted tissue penetration. MENPs should not be confused with conventional magnetic nanoparticles, e.g., superparamagnetic iron oxide nanoparticles (SPIONs). Unlike MENPs, conventional magnetic nanoparticles do not display magnetoelectricity; thus, they cannot directly control local electric fields. It is noteworthy that this feasibility study not only shows the potential of MENPs for the treatment of PDAC but also, due to its field‐driven theranostic capability, potentially for a wide range of cancers. A major hurdle for applying electric fields in biomedical contexts is the rapid screening and dissipation of static or localized fields in conductive biological environments—a phenomenon governed by the nanometer‐scale Debye length of physiological fluid.^[^ ^39^, ^40^, ^41^ ^]^ Consequently, electric fields applied via traditional electrodes or nanoscale sources fail to propagate effectively within biological environments, limiting wireless, precise targeting of therapeutic electric fields. As a result, electric fields delivered by conventional means lack effective propagation and tissue specificity.^[^ ^42^, ^43^ ^]^

MENPs circumvent these barriers by transducing externally applied magnetic fields—which are not attenuated by tissue conductivity—into locally concentrated electric fields at the nanoparticle surface. Their magnetoelectric core–shell design creates polarization at the membrane interface, with minimal field dissipation due to direct contact with the cell membrane. Figure S10 (Supporting Information) illustrates this underlying physics. The surface charge on the nanoparticle's side interfacing with the membrane cannot be screened out, unlike on the opposite, non‐interfacing side, which is immediately screened by ions, in turn leading to maximizing the nanoparticle's field across the membrane. Through this mechanism, MENPs can induce localized, controlled effects at the membrane, possibly including electropermeation, production of reactive oxygen species, and initiation of programmed cell death. The precise mechanisms perhaps overlap with those found in other electric field‐based treatments, such as tumor‐treating fields, rather than proceeding via a single, defined pathway.^[^ ^44^ ^]^

Clinically, MENPs can be administered intravenously, magnetically guided to tumors using weak external fields, and wirelessly activated within the strong static magnetic fields (>1 T) of an MRI scanner. Activation by magnetic fields can produce local electric fields exceeding 10,000 V/cm at the membrane interface on demand, thus providing targeted control of equilibrium conditions at the membrane, also significantly surpassing the molecular‐level electric field thresholds for many of the established biological processes, e.g., membrane permeation, mitotic spindle realignment. Importantly, MENPs also possess reciprocal sensing capabilities through MRI T_2_* or magnetic particle imaging, enabling simultaneous treatment and real‐time imaging—a unique theranostic feature.

In contrast, conventional electric field‐based treatment modalities rely on invasive or superficial electrodes delivering high‐voltage pulses, associated with procedural risks and limited spatial precision. MENPs offer a minimally invasive alternative by transducing alternating or strong static magnetic fields into spatially and temporally controlled membrane electric fields. It is important to note that, unlike conventional superparamagnetic or paramagnetic nanoparticles, due to their magnetoelectric effect, MENPs uniquely enable both induction (“writing”) and sensing (“reading”) of local electric fields in real time—essential for precision, image‐guided cancer theranostics currently unattainable with existing nanomaterials. The concept can be enabled by any MENP configuration as long as the nanoparticles meet certain basic requirements, e.g., having a sufficiently large magnetoelectric coefficient, preferably on the order of 1 V·cm^−1^·Oe^−1^, being adequately dispersed, e.g., through surface hydroxylation, being biocompatible, and others. For the sake of simplicity and the focus on this theranostic application, rather than the MENPs’ materials science, this study was conducted using the most widely used MENP configuration. Particularly, the material system used had a core‐shell configuration with lattice‐matched ferrimagnetic cobalt ferrite (CoFe2O4) core and piezoelectric barium titanate (BaTiO3) shell, creating an efficient magnetoelectric coefficient (≈1 V·cm^−1^·Oe^−1^) and generating electric outputs well beyond clinical IRE thresholds.^[^ ^37^ ^]^ Our hope is that this novel study, showing the theranostic potential of MENPs, can also trigger further interest in the materials side of MENPs, leading to the development of novel, potentially biodegradable compositions.^[^ ^45^, ^46^ ^]^

The efficiency and selectivity of MENP targeting is determined by a combination of magnetoelectricity, magnetic susceptibility, van der Waals orientation, and—most critically—the cancer cell membrane's unique dielectric properties under slow, relatively weak, magnetic steering fields, with the distinction “noticeable” owing to the ME effect. This direct electric‐field‐induced and magnetic‐field‐controlled targeting distinction (cancer specificity) is not provided by conventional magnetic nanoparticles with no ME effect. Indeed, recent theoretical modeling and numerical simulation indicate that it is due to the ME effect that the field‐driven MENP‐membrane interactions can exploit differences in membrane potential and conductivity between malignant and normal cells, allowing MENPs to accumulate at tumor sites even within complex stromal environments.^[^ ^28^ ^]^ This is further augmented by their PEGylation, which balances membrane affinity, dispersion, and biocompatibility, and by protocol design favoring close membrane proximity (which, as shown, is essential for local electric field efficacy). It is possible that in the future, a different surface treatment approach, e.g., increasing the nanoparticles’ hydrophilicity via OH groups, could be pursued to improve their dispersion while maintaining membrane affinity, thus further improving the overall treatment efficacy.^[^ ^47^ ^]^

Systemically administered MENPs, after magnetic targeting and MRI activation in vivo, yielded robust, dose‐dependent tumor ablation (33.3% complete response rate) without observable systemic toxicity or organ pathology, even with repeated dosing and extended follow‐up. The close correlation between intratumoral T_2_* reduction and tumor shrinkage underscores the power of MENPs for direct, quantitative, non‐invasive treatment monitoring—potentially allowing real‐time response‐adaptive therapy. Dose‐response modeling revealed that normalized MENP dose per tumor volume is the best predictor of treatment success, suggesting future clinical regimens should account for baseline tumor burden and may benefit from early imaging biomarker feedback.

Flow cytometry and time‐course studies indicate that MENP‐induced cytotoxicity is mediated predominantly through apoptosis, not necrosis, with cells passing progressively through apoptotic checkpoints. The observed generation of ROS under activation supports the hypothesis that controlled electropermeation and related membrane‐level stress responses—rather than non‐specific tissue injury—underlie this mechanism. This profile suggests a therapeutic window that may allow for precise ablation of malignant tissues while sparing healthy parenchyma, with reduced risk for collateral damage, which is particularly crucial in anatomically complex sites like the pancreas.

Compared to existing local therapies (IRE, thermal ablation, radiofrequency, photodynamic therapy), MENP technology offers a uniquely non‐invasive and remotely controllable solution, integrating treatment and imaging using widely available MRI scanners (Table 1). The favorable toxicity profile highlights the distinction over drug‐based and some other nanoparticle‐based approaches, which often suffer from off‐target immune activation, complex clearance, or persistent tissue retention. Notably, preliminary reports suggest MENPs are rapidly cleared via hepatobiliary and renal pathways,^[^ ^45^ ^]^ and prior animal studies (including those in non‐human primates) report no significant adverse effects at therapeutic dosing.^[^ ^48^ ^]^ Furthermore, the mechanistic selectivity shown here may translate to other solid tumors with abnormal electrophysiological profiles, broadening the platform's utility.

**Table 1:** A comparative analysis of different therapeutic modalities for pancreatic cancer. The therapies are evaluated based on their mechanism of action, invasiveness, spatial specificity, and potential for systemic side effects. The table highlights nanoparticle‐based approaches, such as Magnetoelectric Nanotherapy and Magnetic Thermal Ablation, alongside established treatments like IRE, Radiofrequency Ablation, and both traditional and nanoparticle‐based chemotherapy. This comparison serves to illustrate the distinct advantages of targeted therapies, particularly their potential to enhance treatment efficacy while mitigating the widespread toxicity associated with conventional systemic treatments.

Therapy	Mechanism of Action	Invasiveness	Spatial Selectivity	Toxicity
Magnetoelectric Nanotherapy	Wirelessly activated nanoparticles generate localized electric fields, inducing apoptosis.	**Non‐Invasive** Administered intravenously with external magnetic localization and targeting.	**High**. Activation may be confined to the targeted tumor site by an externally applied magnetic field. MENPs electrically target the unique electrophysiology of the tumor microenvironment. MENPs show cell type specificity to cancer cells over healthy equivalents. Minimal damage to surrounding healthy tissue.	**Minimal**. The therapy is localized and drug‐free, avoiding the systemic toxicity of conventional chemotherapy.
Magnetic Thermal Ablation doi: https://doi.org/10.7150/thno.40805	Magnetic nanoparticles heat up under an alternating magnetic field, causing hyperthermia that kills cancer cells.	**Non‐Invasive to Minimally Invasive**. Requires direct injection into the tumor or intravenous delivery.	**Moderate**. The heat is generated where the magnetic nanoparticles are located, allowing for targeted ablation. This is reliant on targeting efficacy and suffers from potential “heat‐sink” effects.	**Minimal to moderate**. Side effects are generally localized to the treatment area, though some transient effects like nausea or vomiting have been reported.
IRE https://doi.org/10.1038/s41467‐019‐08782‐1	Uses high‐voltage electrical pulses to create permanent “nanopores” in cell membranes, leading to apoptosis and cell death.	**Minimally Invasive to Invasive**. Requires either percutaneous or surgical insertion of multiple electrodes into the tumor.	**High**. Preserves delicate structures like blood vessels and bile ducts, which is a major advantage over thermal ablation.	**Minimal to severe**. The effects are localized, and it is a non‐thermal process, but it is not completely without risk of severe adverse cardiac events and significant hemorrhage.
Radiofrequency Ablation https://doi.org/10.1038/sj.bjc.6602582	Uses a probe to deliver radiofrequency energy, generating heat that causes thermal coagulative necrosis.	**Minimally Invasive to Invasive**. Requires percutaneous or surgical insertion of a probe.	**Moderate**. Can be less specific than IRE due to heat spread and the “heat‐sink” effect near blood vessels, which can lead to incomplete ablation.	**Minimal to moderate**. Effects are localized, but can cause localized pain and hemorrhage.
Traditional Chemotherapy https://doi.org/10.1016/j.gendis.2022.02.007	Systemic drugs interfere with DNA, RNA, and protein synthesis or disrupt mitosis, killing rapidly dividing cells.	**Non‐Invasive**. Administered intravenously, orally, or in a combination of both.	**Poor**. Kills all rapidly dividing cells, not just cancer cells.	**Moderate to High**. Damages healthy cells (e.g., hair follicles, bone marrow, digestive tract lining), leading to widespread side effects like hair loss, nausea, and immune suppression.
Nanoparticle‐based Chemotherapy doi: https://doi.org/10.3389/fmolb.2020.00193	Encapsulates chemotherapy drugs in nanoparticles to improve delivery to the tumor.	**Non‐Invasive**. Administered intravenously.	**Moderate**. Uses the EPR effect to passively accumulate in tumors. This can improve targeting over traditional chemo but is not perfectly specific.	**Reduced**. The encapsulation can reduce systemic exposure and off‐target toxicity compared to traditional chemotherapy, but side effects are still common.

While these findings are compelling, several limitations warrant acknowledgement. The relationship between MENP concentration and exact intratumoral distribution, inferred through MRI signal modulation, remains to be directly confirmed by future, more detailed, quantitative biodistribution studies; future work should employ more comprehensive histologic or spectroscopic mapping to further validate this link. In addition, the contribution of immune‐mediated responses, long‐term effects beyond the study window, and scalability to larger animal models and ultimately humans would need to be determined. The precise biophysical parameters (field strengths, timing, exposure frequency) for optimal efficacy—and the interplay with variable tumor microenvironments—require further optimization.

In practice, clinical translation will demand refined manufacturing, validated regulatory protocols, and rigorous toxicology in multiple preclinical models. Additionally, future research should address the potential immunomodulatory or synergistic roles with immunotherapy (as is emerging for IRE), combination scheduling with standard‐of‐care regimens, and the refinement of early MRI‐based biomarkers for treatment planning.

Taken together, this study demonstrates that MENPs can be used to achieve targeted, minimally invasive, externally controlled ablation and real‐time monitoring of solid tumors, without using any bioreagents, instead relying on precise control of electric and magnetic fields, potentially at the molecular level that underlies fundamental biological mechanisms. In layman's terms, MENPs allow to reap the undeniable benefits of relatively strong electric fields without the common devastating side effects due to the unavoidable off‐targeting of the conventional treatment, because MENPs generate the same order magnitude electric fields in extremely localized target sites only, and they do it wirelessly in response to the application of a magnetic field. By combining high‐specificity targeting, potent local cytotoxicity, and integrated imaging, MENPs establish a strong foundation as a novel theranostic platform in oncology. If validated in larger models and human studies, this approach could improve the outlook of cancer care, unlocking a new dynasty of precision therapies with feedback‐based adaptation—allowing for safer, more effective, highly personalized treatment of PDAC and other aggressive malignancies.

## 4 Experimental Section

### MENP Synthesis

The MENPs used in this study were synthesized in a similar process as has been previously described, however, modified to promote anisotropic growth.^[^ ^49^ ^]^ MENPs were synthesized from the following chemicals: cobalt(II) nitrate hexahydrate (Co(NO_3_)_2_•6H_2_O) (Sigma–Aldrich, St Louis, MO), iron(III) nitrate nonahydrate (Fe(NO_3_)_2_•9H_2_O) (Sigma–Aldrich), sodium hydroxide (NaOH; Sigma–Aldrich), barium carbonate (BaCO_3_) (ThermoFisher Scientific, Waltham, MA), titanium(IV) isoproproxide (Ti[OCH(CH_3_)_2_]_4_) (Sigma–Aldrich), citric acid (HOC(COOH)(CH_2_COOH)_2_) (Sigma–Aldrich), and ethanol (>99.7%) (Millipore Sigma, Burlington, MA). All reagents were used without further purification.

The cobalt ferrite cores were fabricated via a coprecipitation process using metal salts of cobalt and iron, with sodium hydroxide as the precipitating agent. The barium titanate shells were formed on the cobalt ferrite cores at a 1:2 (core: shell) stochiometric ratio using a modified sol‐gel and auto‐combustion process. Complete synthesis protocols and detailed procedural steps are provided in the Supporting Information.

### Nanoparticle Characterization

The magnetic properties of the nanoparticles were measured using a MicroMag 2900 Alternating Gradient Magnetometer (Lakeshore, Carson, CA). M‐H loops were derived from either ±0.5 or ±1 T sweeps to achieve saturation. The size and shape of the nanoparticles were verified using a multimode atomic force microscope (Bruker, Billerica, MA), a magnetic force microscope (Bruker), a high‐resolution transmission electron microscope (JEOL JEM‐2100 Plus with a 200 kV LaB6 electron source; Jeol USA, Peabody, MA), and dynamic light scattering measurements. The elemental composition was independently confirmed through energy‐dispersive spectroscopy and x‐ray diffraction analyses.^[^ ^15^, ^25^ ^]^ Results of the characterization are in Figure S1 (Supporting Information).

### Dye Degradation Tests

To assess reactive oxygen species (ROS) generation by MENPs under magnetic stimulation, a diluted Trypan blue solution (0.04%) was incubated with MENPs (5 mg mL^−1^) following a method outlined previously in the literature.^[^ ^50^ ^]^ Samples were divided into two groups: one exposed to probe sonication alone and another exposed to 1 kHz, 0.025 T alternating magnetic fields during sonication for 1 h. Following treatment, MENPs were removed by centrifugation, and supernatants were diluted 1:4 with deionized water. Absorbance at ∼580 nm was measured via UV–vis spectrophotometry to evaluate dye degradation. Each condition was performed in triplicate.

### Murine Pancreatic Ductal Adenocarcinoma Culture

In vitro and in vivo experiments were conducted using the UN‐KPC‐961 pancreatic cell line. The exact RRID identifier was missing because this cell line was obtained via a material transfer agreement from Dr. Surinder K Batra (University of Nebraska Medical Center, Omaha, NE). Cells were maintained and cultured according to their suggested protocols using DMEM:F12 supplemented with 10% heat‐inactivated fetal bovine serum (FBS) and penicillin‐streptomycin (100 units/mL–100 mg mL^−1^). All cells were maintained in a contamination‐free environment under standard tissue culture conditions at 37 °C and 5% CO2 and were tested and kept mycoplasma‐free. All steps were performed under sterile conditions in a biosafety cabinet, shown briefly in Figure S2 (Supporting Information). The specific choice for the cell line reflected our goal to launch pancreatic cancer applications of this novel concept. No conclusion remarks are being affected.

### Nanoparticle Suspension Preparations

To enhance colloidal stability, biocompatibility, and membrane targeting, MENPs were coated with polyethylene glycol (PEG, MW 600 Da). MENPs were dispersed in deionized water (1:1 ratio) and probe‐sonicated for 2 h (3 s on/3 s off cycle, 20% amplitude). Liquid PEG was added at ≈8% volume, and the mixture was sonicated for an additional 2 h under the same conditions. The PEGylated nanoparticles were washed three times by centrifugation (3000 × g, 7 min) and resuspended in deionized water at desired concentrations for in vivo use. Prior to injection, MENP suspensions were magnetized using a 0.15 T permanent magnet and sonicated briefly (≈30 min) to ensure optimal dispersion.

### MRI Scans

All the in vitro and in vivo experiments were conducted with a horizontal 7 T Biospec MRI (Bruker, Billerica, MA) using a 35 mm birdcage coil (Doty Scientific, Richland County, SC). Mapping studies were always performed in the following order: T_2_* first, T_2_ second, and T_1_ last. Gradient‐echo sequences (GES) were used to acquire T_2_* images, while Rapid Acquisition with Relaxation Enhancement (RARE) sequences were used to acquire T_1_ and T_2_ images. Mapping sessions took 45 min to complete 1 set of all mapping scans.

For T_2_*, a gradient‐echo sequence was used to acquire 10 echoes, starting at 3.5 ms, with a 5 ms echo spacing. Images were acquired with a flip angle of 50°, a matrix size of 256 × 256 pixels, 14 slices, and 2 averages. The resulting T_2_* relaxation time maps were created by fitting voxel‐wise signal intensity data from these gradient‐echo images to an exponential decay model. These maps were used to measure the average intratumoral T_2_* relaxation times at baseline and on day 2.

For T_1_ and T_2_, a RARE sequence was used with different parameter sets to optimize data acquisition. T_1_ images were acquired using 6 TRs with a RARE factor of 2, while T_2_ images were acquired using five echoes. The matrix size was 256 × 128 pixels, with 14 slices. T_1_ relaxation maps were generated by fitting signal intensity data from the six TRs, and T_2_ relaxation maps were created using voxel‐wise fits of the signal intensities from the five echoes. The T_1_ and T_2_ maps were used to identify tumor volumes accurately.

This combination of imaging approaches allowed for both precise anatomical visualization and quantitative measurement of tumor relaxation times, providing robust data for evaluating intratumoral characteristics and changes over time.

### In Vitro Cell Death and Flow Cytometry

Murine PDAC cells (KPC‐961) were cultured under standard conditions (DMEM/F12 with 10% FBS and 1% penicillin‐streptomycin) and maintained mycoplasma‐free. Six million cells in 1 mL media were aliquoted into 5 mL tubes (*N* = 3 MENP‐treated, *N* = 3 untreated controls). Cells were subjected to 7 T MRI scans (T_1_, T_2_, and T_2_* mapping) with the MENP‐treated group exposed to MRI activation to induce local electric fields via the magnetoelectric effect. Following MRI, samples were immediately analyzed by flow cytometry using Annexin V‐FITC and propidium iodide staining to assess apoptosis and necrosis. Approximately 1 million cells per sample were incubated with staining reagents according to the manufacturer's protocol (BioLegend Annexin V/PI kit). Data acquisition was performed on a FACSort cytometer and analyzed using FlowJo software.

### Murine Tumor Models

Female and male C57BL/6 mice (6–8 weeks old) were inoculated subcutaneously with 1×10^6^ KPC‐961 cells in 100 µL PBS on the right flank. Mice were randomly assigned to treatment cohorts after tumors reached ≈90–120 mm^3^. All procedures were approved by the Institutional Animal Care and Use Committee (IACUC) (protocol IS00010076).

Tumor growth was monitored by calipers and 7 T MRI scans at baseline, day 2, and day 7. Tail vein injections of MENPs or controls were administered under anesthesia, with magnetic targeting via 0.15T neodymium magnets placed over tumors. Tumor volumes and MRI relaxation times were quantified using 3D Slicer, with correction for motion artifacts, with a 1 mm contraction was performed from the edges of the tumor.

The MRI scans performed after day 7, as well as all scans conducted on the non‐activated MENP mouse cohort, utilized a Turbo Spin Echo Rapid Acquisition with Relaxation Enhancement (TurboRARE) sequence. The imaging parameters included a TR of 4000 ms and an echo time (TE) of 48 ms.

Animals were euthanized at study endpoints or when tumors exceeded size limits (tumor volume ≥1,000 mm^3^). Tissue samples were collected for histopathological analysis.^[^ ^51^, ^52^ ^]^ Details of imaging sequences, biopsy processing, and histopathology scoring are provided in Supporting Information.

### Statistical Analysis

Statistical analyses were performed using SPSS v29 (IBM). Data normality was assessed by Shapiro‐Wilk test and Q‐Q plots. Parametric tests (Student's *t*‐test, ANOVA with LSD post‐hoc) were applied to normally distributed data; non‐parametric tests (Mann‐Whitney U, Kruskal‐Wallis with Dunn's post‐hoc) were used for non‐normal data. Multiple comparisons were corrected using Bonferroni adjustments where appropriate. Effect sizes (r‐values) were calculated from standardized test statistics.

In vitro cell death was compared by unpaired t‐tests. Dye degradation was analyzed using Friedman tests with Dunn's post‐hoc. In vivo tumor volume and T_2_* relaxation outcomes were expressed as percentage change from baseline and analyzed accordingly. Two‐way ANOVA tested interactions between dose and baseline tumor volume. ROC curve analyses determined dose thresholds predictive of therapeutic efficacy. Sex‐based analyses were conducted with Mann‐Whitney U tests. Statistical significance was set at *p* < 0.05.

## Conflict of Interest

In 2015, Ping Liang and Sakhrat Khizroev co‐founded a biotech company, Cellular Nanomed, to create the first wireless brain‐computer interface (BCI) based on magnetoelectric nanoparticles (MENPs). The nanoparticles used in this study were developed as a part of the DARPA Next‐generation Non‐surgical Neurotechnology (N3) program contract to Cellular Nanomed.

## Supporting information



Supporting Information

## Data Availability

The data that support the findings of this study are available in the supplementary material of this article.

## References

[1] R. L. Siegel , K. D. Miller , N. S. Wagle , A. Jemal , CA Cancer J. Clin. 2023, 73, 17. 36633525 10.3322/caac.21763

[2] T. Kotnik , L. Rems , M. Tarek , D. Miklavčič , Annu. Rev. Biophys. 2019, 48, 63. 30786231 10.1146/annurev-biophys-052118-115451

[3] A. R. Deipolyi , A. Golberg , M. L. Yarmush , R. S. Arellano , R. Oklu , Diagn. Interv. Radiol. Ank. Turk. 2014, 20, 147. 10.5152/dir.2013.13304 PMC4463294 24412820

[4] H. J. Scheffer , A. G. M. Stam , B. Geboers , L. G. P. H. Vroomen , A. Ruarus , B. De Bruijn , M. P. van den Tol , G. Kazemier , M. R. Meijerink , T. D. de Gruijl , OncoImmunology 2019, 8, 1652532. 31646081 10.1080/2162402X.2019.1652532 PMC6791414

[5] M. C. De Grandis , V. Ascenti , C. Lanza , G. Di Paolo , B. Galassi , A. M. Ierardi , G. Carrafiello , A. Facciorusso , M. Ghidini , Int. J. Mol. Sci. 2023, 24, 12681. 37628865 10.3390/ijms241612681 PMC10454061

[6] J. Zhao , X. Wen , L. Tian , T. Li , C. Xu , X. Wen , M. P. Melancon , S. Gupta , B. Shen , W. Peng , C. Li , Nat. Commun. 2019, 10, 899. 30796212 10.1038/s41467-019-08782-1 PMC6385305

[7] A. Rodzinski , R. Guduru , P. Liang , A. Hadjikhani , T. Stewart , E. Stimphil , C. Runowicz , R. Cote , N. Altman , R. Datar , S. Khizroev , Sci. Rep. 2016, 6, 20867. 26875783 10.1038/srep20867 PMC4753509

[8] M. Nair , R. Guduru , P. Liang , J. Hong , V. Sagar , S. Khizroev , Nat. Commun. 2013, 4, 1707. 23591874 10.1038/ncomms2717

[9] K. Yue , R. Guduru , J. Hong , P. Liang , M. Nair , S. Khizroev , G. Magneto‐Forloni , PLoS One 2012, 7, 44040. 10.1371/journal.pone.0044040 PMC3434207 22957042

[10] K. L. Kozielski , A. Jahanshahi , H. B. Gilbert , Y. Yu , Ö. Erin , D. Francisco , F. Alosaimi , Y. Temel , M. Sitti , Sci. Adv. 2021, 7, abc4189. 10.1126/sciadv.abc4189 PMC7806222 33523872

[11] T. Nguyen , J. Gao , P. Wang , A. Nagesetti , P. Andrews , S. Masood , Z. Vriesman , P. Liang , S. Khizroev , X. Jin , Neurotherapeutics 2021, 18, P2091. 10.1007/s13311-021-01071-0 PMC8609092 34131858

[12] S. Fiocchi , E. Chiaramello , A. Marrella , M. Bonato , M. Parazzini , P. Ravazzani , J. Neural Eng. 2022, 19, 056020. 10.1088/1741-2552/ac9085 36075197

[13] E. Zhang , M. Abdel‐Mottaleb , P. Liang , B. Navarrete , Y. A Yildirim , M. A Campos , I. T. Smith , P. Wang , B. Yildirim , L. Yang , S. Chen , I. Smith , G. Lur , T. Nguyen , X. Jin , B. R. Noga , P. Ganzer , S. Khizroev , Brain Stimulat. 2022, 15, 1451. 10.1016/j.brs.2022.10.004 36374738

[14] F. Mushtaq , H. Torlakcik , Q. Vallmajo‐Martin , E. C. Siringil , J. Zhang , C. Röhrig , Y. Shen , Y. Yu , X.‐Z. Chen , R. Müller , B. J. Nelson , S. Pané , Appl. Mater Today 2019, 16, 290.

[15] J. Jang , C. B. Park , Sci. Adv. 2022, 8, abn1675. 10.1126/sciadv.abn1675 PMC9094672 35544560

[16] Y. J. Kim , N. Kent , E. Vargas Paniagua , N. Driscoll , A. Tabet , F. Koehler , E. Malkin , E. Frey , M. Manthey , A. Sahasrabudhe , T. M. Cannon , K. Nagao , D. Mankus , M. Bisher , G. de Nola , A. Lytton‐Jean , L. Signorelli , D. Gregurec , P. Anikeeva , Nat. Nanotechnol. 2025, 20, 121. 39394431 10.1038/s41565-024-01798-9 PMC11750723

[17] E. Zhang , M. Shotbolt , C.‐Y. Chang , A. Scott‐Vandeusen , S. Chen , P. Liang , D. Radu , S. Khizroev , Brain Stimul. 2024, 17, 1005. 39209064 10.1016/j.brs.2024.08.008

[18] R. C. G. Martin , D. Kwon , S. Chalikonda , M. Sellers , E. Kotz , C. Scoggins , K. M. McMasters , K. Watkins , Ann Surg. 2015, 262, 486. 26258317 10.1097/SLA.0000000000001441

[19] R. Guduru , P. Liang , C. Runowicz , M. Nair , V. Atluri , S. Khizroev , Sci. Rep. 2013, 16, 2953. 10.1038/srep02953 PMC3797424 24129652

[20] C. Jiang , Z. Qin , J. Bischof , Ann. Biomed. Eng. 2014, 42, 193. 23949655 10.1007/s10439-013-0882-7

[21] I. Bok , I. Haber , X. Qu , A. Hai , Sci. Rep. 2022, 12, 8386. 35589877 10.1038/s41598-022-12303-4 PMC9120189

[22] A. Kaushik , J. Rodriguez , D. Rothen , V. Bhardwaj , R. D. Jayant , P. Pattany , B. Fuentes , H. Chand , N. Kolishetti , N. El‐Hage , K. Khalili , N. S. Kenyon , M. Nair , ACS Appl. Bio. Mater. 2019, 2, 4826. 10.1021/acsabm.9b00592 PMC10077812 35021482

[23] A. Nagesetti , A. Rodzinski , E. Stimphil , T. Stewart , C. Khanal , P. Wang , R. Guduru , P. Liang , I. Agoulnik , J. Horstmyer , S. Khizroev , Sci. Rep. 2017, 7, 1610. 28487517 10.1038/s41598-017-01647-x PMC5431629

[24] S. Khizroev , Cold Spring Harb. Perspect. Med. 2019, 9, a034207. 30291147 10.1101/cshperspect.a034207 PMC6671931

[25] E. Zhang , P. Liang , Y. A. Yildirim , S. Chen , M. Abdel‐Mottaleb , M. Shotbolt , Z. Ramezani , J. Tian , V. Andre , S. Khizroev , IEEE Trans. Magn. 2023, 59, 1.

[26] R. Guduru , P. Liang , M. Yousef , J. Horstmyer , S. Khizroev , Bioelectron Med. 2018, 4, 10. 32232086 10.1186/s42234-018-0012-9 PMC7098259

[27] S. Caspani , R. Magalhães , J. P. Araújo , C. T. Sousa , Mater Basel Switz. 2020, 13, 2586. 10.3390/ma13112586 PMC7321635 32517085

[28] M. Shotbolt , J. Bryant , P. Liang , S. Khizroev , Adv. Theory Simul. 2025, 00739.

[29] E. Stimphil , A. Nagesetti , R. Guduru , T. Stewart , A. Rodzinski , P. Liang , S. Khizroev , Appl. Phys. Rev. 2017, 4, 021101.

[30] X. Zhang , G. Ma , W. Wei , NPG Asia Mater. 2021, 13, 52.

[31] S. Zhang , H. Gao , G. Bao , ACS Nano. 2015, 9, 8655. 26256227 10.1021/acsnano.5b03184 PMC5681865

[32] P. Liang , Methods for killing cancer cells and cellular imaging using magneto‐electric nano‐particles and external magnetic field, US Patent No 10,188,731 B2, issued January 29, 2019.

[33] M. Shotbolt , E. Zhang , E. Zhu , W. El‐Rifai , J. Bryant , P. Liang , S. Khizroev , Cancer Res. 2024, 84, 490.

[34] P. Martins , R. Brito‐Pereira , S. Ribeiro , S. Lanceros‐Mendez , C. Ribeiro , Nano Energy 2024, 126, 109569.

[35] I. T. Smith , E. Zhang , Y. A. Yildirim , M. A. Campos , M. Abdel‐Mottaleb , B. Yildirim , Z. Ramezani , V. Louise Ramezani , A. Scott‐Vandeusen , P. Liang , S. Khizroev , WIREs Nanomed. Nanobiotechn. 2022, 15, 1849.

[36] V. Andre , M. Abdel‐Mottaleb , M. Shotbolt , S. Chen , Z. Ramezini , E. Zhang , S. Conlan , O. Telisman , P. Liang , J. M. Bryant , R. Chomko , S. Khizroev , Nanoscale Horiz. 2025, 10, 699. 39898755 10.1039/d4nh00560k PMC11789716

[37] P. Wang , E. Zhang , D. Toledo , I. T. Smith , B. Navarrete , N. Furman , A. F. Hernandez , M. Telusma , D. McDaniel , P. Liang , S. Khizroev , Nano Lett. 2020, 20, 5765. 32639738 10.1021/acs.nanolett.0c01588

[38] E. C. Stoner , E. P. Wohlfarth , IEEE Trans Magn. 1991, 27, 3475.

[39] V. Kesler , B. Murmann , H. T. Soh , ACS Nano. 2020, 14, 16194. 33226776 10.1021/acsnano.0c08622 PMC7761593

[40] J. V. Raimondo , R. J. Burman , A. A. Katz , C. J. Akerman , Front. Cell Neurosci. 2015, 9, 419. 26539081 10.3389/fncel.2015.00419 PMC4612498

[41] M. Levin , Semin. Cell Dev. Biol. 2009, 20, 543. 19406249 10.1016/j.semcdb.2009.04.013 PMC2706303

[42] B. Geboers , H. J. Scheffer , P. M. Graybill , A. H. Ruarus , S. Nieuwenhuizen , R. S. Puijk , P. M. van den Tol , R. V. Davalos , B. Rubinsky , T. D. de Gruijl , D. Miklavčič , M. R Meijerink , Gene Electrotransfer, Electrof. Electroimmunotherapy. Radiol. 2020, 295, 254. 10.1148/radiol.2020192190 32208094

[43] M. Yang , W. J. Brackenbury , Front. Physiol. 2013, 4, 00185. 10.3389/fphys.2013.00185 PMC3713347 23882223

[44] R. J. DeBerardinis , N. S. Chandel , Sci. Adv. 2016, 2, 1600200. 10.1126/sciadv.1600200 PMC4928883 27386546

[45] A. Hadjikhani , A. Rodzinski , P. Wang , A. Nagesetti , R. Guduru , P. Liang , C. Runowicz , S. Shahbazmohamadi , S. Khizroev , Nanomedicine 2017, 12, 1801. 28705034 10.2217/nnm-2017-0080 PMC5551528

[46] B. Fadeel , G.‐B. AE , Adv. Drug Delivery Rev. 2010, 62, 362. 10.1016/j.addr.2009.11.008 19900497

[47] D. Kim , I. Efe , H. Torlakcik , A. Terzopoulou , A. Veciana , E. Siringil , F. Mushtaq , C. Franco , D. von Arx , S. Sevim , J. Puigmartí‐Luis , B. Nelson , N. A. Spaldin , C. Gattinoni , X.‐Z. Chen , S. Pané , Adv. Mater. 2022, 34, 2110612. 10.1002/adma.202110612 35276030

[48] A. Kaushik , J. Rodriguez , D. Rothen , V. Bhardwaj , R. D. Jayant , P. Pattany , B. Fuentes , H. Chand , N. Kolishetti , N. El‐Hage , K. Khalili , N. S. Kenyon , M. Nair , ACS Appl. Bio. Mater. 2019, 2, 4826. 10.1021/acsabm.9b00592 PMC10077812 35021482

[49] A. Marrella , G. Suarato , S. Fiocchi , E. Chiaramello , M. Bonato , M. Parazzini , P. Ravazzani , Front. Bioeng. Biotechnol. 2023, 11, 1219777. 37691903 10.3389/fbioe.2023.1219777 PMC10485842

[50] F. Mushtaq , X. Chen , H. Torlakcik , C. Steuer , M. Hoop , E. C. Siringil , X. Marti , G. Limburg , P. Stipp , B. J. Nelson , S. Pané , Adv. Mater. 2019, 31, 1901378. 10.1002/adma.201901378 31045284

[51] C. W. Schmidt , Environ. Health Perspect. 2014, 122, A76. 24583717 10.1289/ehp.122-A76 PMC3948027

[52] A. Fedorov , R. Beichel , J. Kalpathy‐Cramer , J. Finet , J.‐C. Fillion‐Robin , S. Pujol , C. Bauer , D. Jennings , F. Fennessy , M. Sonka , J. Buatti , S. Aylward , J. V. Miller , S. Pieper , R. Kikinis , Magn. Reson. Imaging. 2012, 30, 1323. 22770690 10.1016/j.mri.2012.05.001 PMC3466397

