# Supplementary material for: Magnetoelectric Nanotherapy Achieves Complete Tumor Ablation and Prolonged Survival in Pancreatic Cancer Murine Models
Source: Adv Sci (Weinh). 2025 Nov 3;13(9):e17228. doi: 10.1002/advs.202517228 (PMC12904064; doi:10.1002/advs.202517228)
Supplement: Supplementary file 1 — Supporting Information [file ADVS-13-e17228-s001.docx]

# Supplementary Materials

**MENP Synthesis**

MENPs were synthesized from the following chemicals: cobalt(II) nitrate hexahydrate, iron(III) nitrate nonahydrate, sodium hydroxide, barium carbonate, titanium(IV) isopropoxide, citric acid, and ethanol. All reagents were used without further purification.

**Cobalt Ferrite Core Fabrication**

The cobalt ferrite cores were fabricated via a coprecipitation process. In a typical synthesis, 100 mg of cobalt nitrate and 278 mg of iron nitrate were dissolved with constant stirring into separate beakers containing 20 mL of deionized (DI) water. The beakers were heated to near 90 °C and then mixed together. A 3M sodium hydroxide aqueous solution was added until the mixture reached a pH of 13, at which point precipitation began. The reaction was allowed to proceed for 1 hour to reach a particle size of 25 to 30 nm. The solution was then cooled, and the cobalt ferrite nanoparticles were magnetically separated. The nanoparticles were washed twice in DI water and once in ethanol before being dried overnight on an 80 °C hotplate.

**Barium Titanate Shell Formation**

The barium titanate shells were formed on the cobalt ferrite cores at a 1:2 (core:shell) stoichiometric ratio using a modified sol-gel and auto-combustion process. For synthesis, 50 mg of the prepared cobalt ferrite cores were mixed in 20 mL of DI water with 1000 mg of citric acid. This beaker was then probe sonicated for 2 hours to fully disperse the cores. In a separate beaker, 88 mg of barium carbonate was mixed with 20 mL of DI water, and 126 μL of titanium isopropoxide was added.


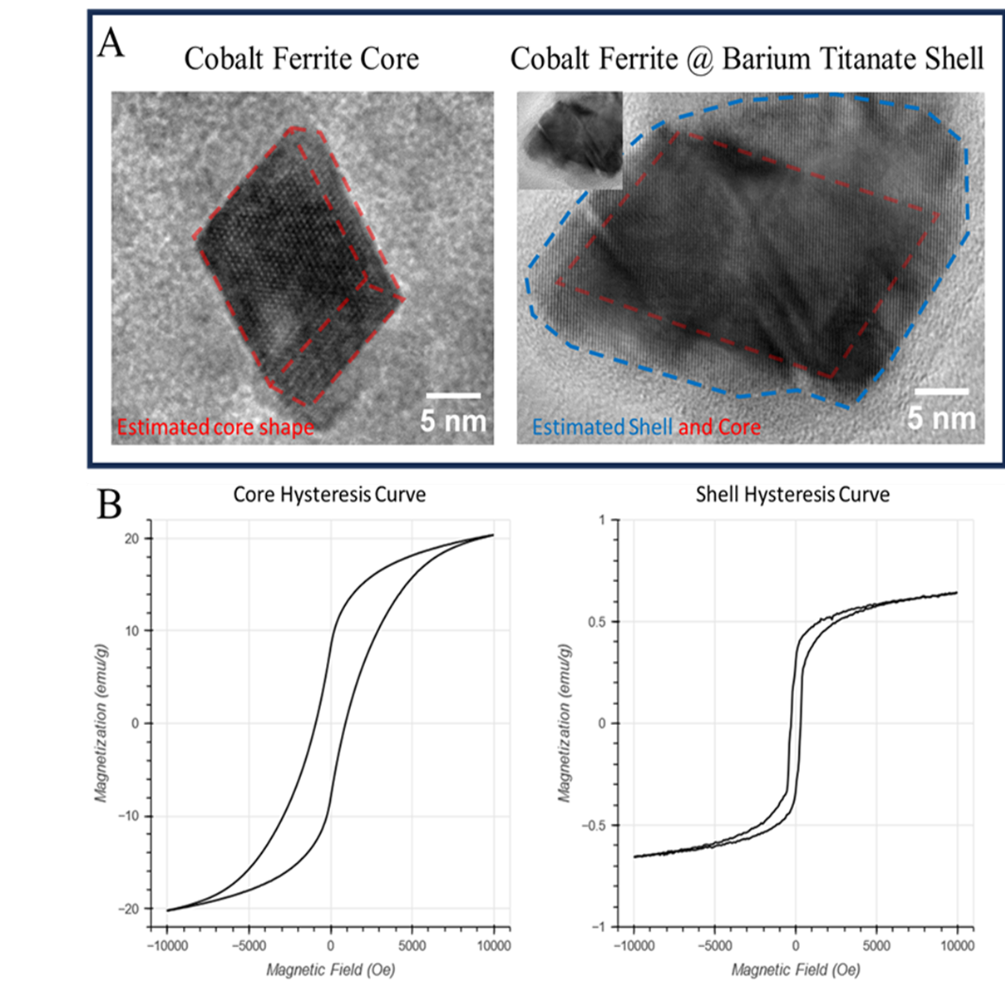


**Fig. S1: MENP TEM Imaging and M-H loop characterizations.** (**A) MENP high resolution-TEM Imaging**. Images of the magnetostrictive cobalt-ferrite-based inverse-spinel ferrimagnetic core of a rectangular prism shape and the core-shell nanostructure with the piezoelectric barium-titanite-based perovskite shell enclosing the magnetostrictive core for the MENPs. The core and the shell are highlighted by red and blue broken lines, respectively. (**B)** **M-H loop characterizations.** M-H loops of the core nanoparticles and the core-shell nanoparticles, measured with AGM Lakeshore Nanomag 2900 (Lake Shore Cryotronics, Westerville, OH, USA).


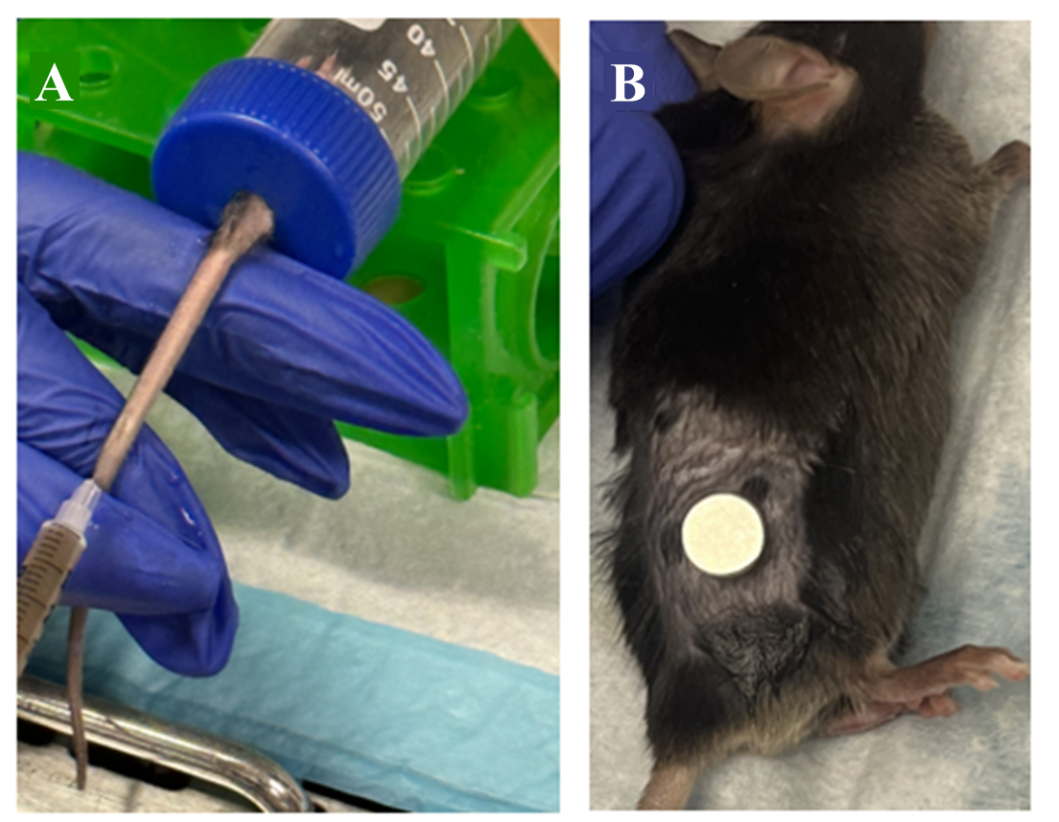


**Fig. S2: Photo examples of tail vein injection and targeting magnet placement. (A) Tail vein injection.** The mice were placed within a 50 mL Eppendorf conical tube with a small hole placed through the top to pull the tail through. Every mouse in the study received a 300 µL tail vein injection in between the M0 and M1 timepoints. **(B)** **Neodymium magnet placement.** The magnets (dimensions = 5.64 mm height x 4.0 mm radius) were placed over right sided flank tumors and taken off immediately before MRI session on day 2. Magnet adhesion was accomplished with a small application of VetBond Tissue Adhesive (3M, Saint Paul, MN, USA) to the flank tumor and subsequent placement of the neodymium magnet. These magnets were both placed and removed when the mice were anesthetized with 2% isoflurane in O_2_ to allow for adhesive to dry in place and for gentle manual traction removal.


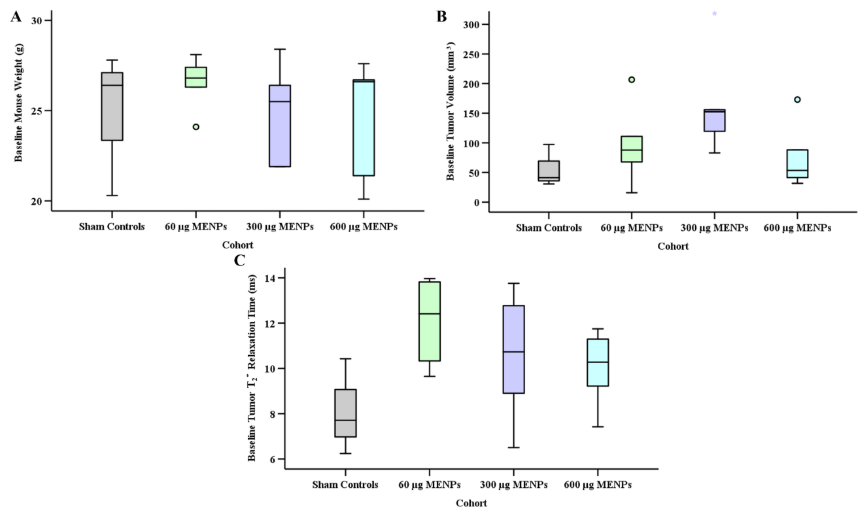


**Fig. S3: Baseline mouse weight and tumor characteristics in the pilot study.** **(A) Baseline weights across cohorts.** No significant differences were observed between cohorts (One-way ANOVA, F = 0.194, df = 3, *P* = 0.899). **(B) Baseline tumor volumes across cohorts.** Similarly, no significant differences were detected (One-way ANOVA, F = 2.031, df = 3, *P* = 0.156). **(C) Baseline T_2_^*^ relaxation times.** No baseline differences between cohorts (One-way ANOVA, F = 1.955, df = 3, *P* = 0.167). Significant associations are indicated as follows: * for *P* < 0.05, ** for *P* < 0.01, and *** for *P* < 0.001. Abbreviations: ANOVA: analysis of variance; KW: Kruskal-Wallis; MENP: magnetoelectric nanoparticle.


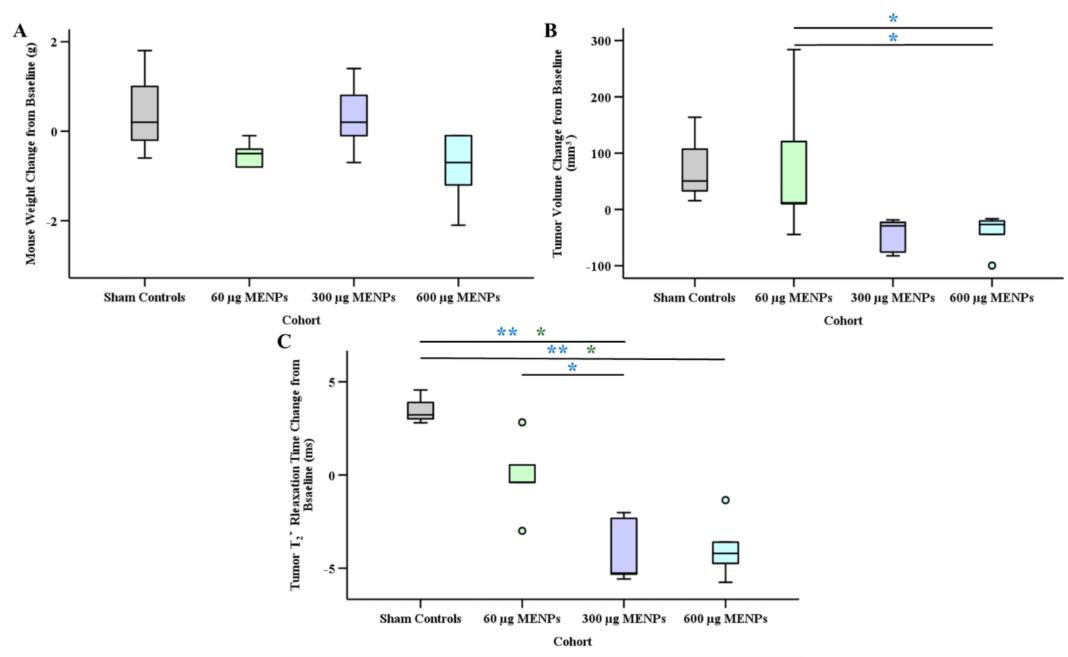


**Fig. S4: Absolute Change in mouse weight and tumor characteristics from baseline in the pilot study.** **(A) Change in weight from baseline to day 7 across cohorts.** No significant differences were observed (One-way ANOVA, F = 2.782, df = 3, *P* = 0.080). **(B) Change in tumor volume from baseline to day 7 across cohorts.** MENP doses of 300 µg and 600 µg showed significant reductions compared to 60 µg, but not sham controls, highlighting limitation of non-normalized data (One-way ANOVA, F = 3.377, df = 3, *P* = 0.049). **(C) Change in T_2_^*^ relaxation times from baseline to day 7 across cohorts.** Doses of 300 µg and 600 µg MENPs demonstrated adjusted significance as compared to sham controls (KW, H = 11.910, df = 3, *P* = 0.008). Significant associations are indicated as follows: * for *P* < 0.05, ** for *P* < 0.01, and *** for *P* < 0.001. Blue asterisks indicate unadjusted (Dunn’s & LSD) significance, and green asterisks indicate adjusted (Bonferroni’s) significance. Abbreviations: ANOVA: analysis of variance; KW: Kruskal-Wallis; MENP: magnetoelectric nanoparticle.


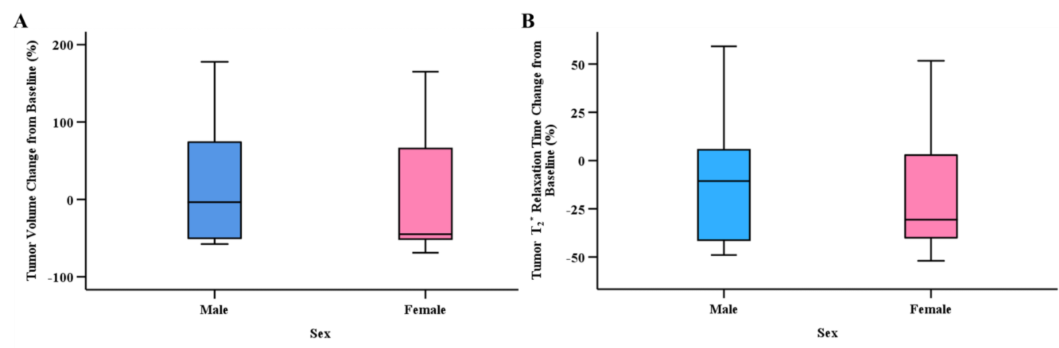


**Fig. S5: Sex-based differences in pilot study. (A) Relative change in tumor volumes from baseline, stratified by sex.** No significant differences were observed between male and female mice (Mann-Whitney U test, U = 31.0, *P* = 0.424; exact *P* = 0.460). **(B) Relative change in tumor T_2_^*^ relaxation time from baseline, stratified by sex.** Similarly, no significant differences were observed (Mann-Whitney U test, U = 35.0, *P* = 0.657; exact *P* = 0.696). Significant associations are indicated as follows: * for *P* < 0.05, ** for *P* < 0.01, and *** for *P* < 0.001.


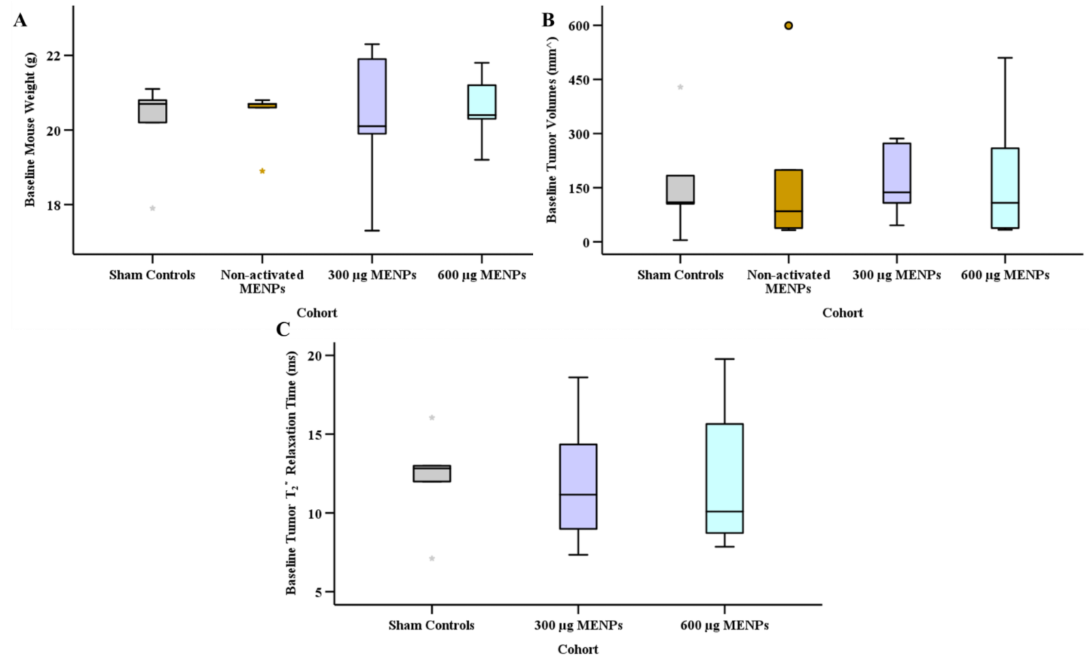


**Fig. S6:** **Fig. S6: Baseline characteristics of mouse cohorts in the confirmatory study. (A) Baseline weights across cohorts.** No significant differences were observed among the groups (KW test, N = 22, H = 0.154, df = 3, *P* = 0.985). **(B) Baseline tumor volumes across cohorts.** Similarly, no significant differences were detected (KW test, N = 22, H = 0.585, df = 3, *P* = 0.900). **(C) Baseline T_2_^*^ relaxation times across cohorts.** Analysis revealed no significant differences in T_2_^*^ relaxation times between groups (One-way ANOVA test, F = 0.006, df = 2, *P* = 0.994). Significant associations are indicated as follows: * for *P* < 0.05, ** for *P* < 0.01, and *** for *P* < 0.001.


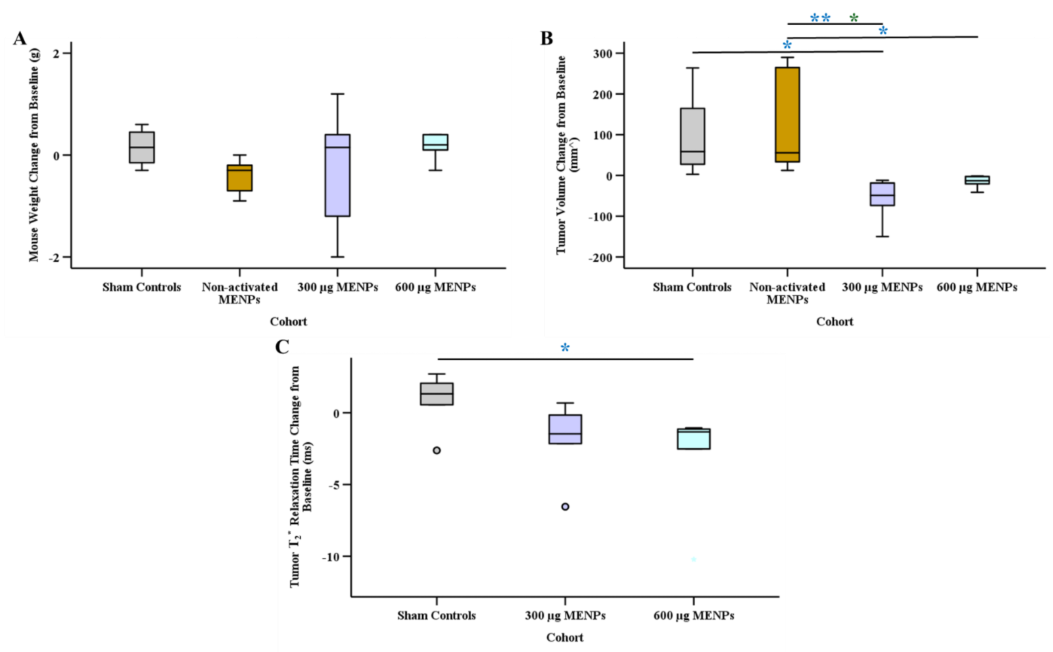


**Fig. S7: Absolute Change in mouse weight and tumor characteristics from baseline in the pilot study. (A) Change in weight from baseline to day 7 across cohorts.** Analysis revealed no significant differences across cohorts (One-way ANOVA test, F = 2.782, df = 3, *P* = 0.080). **(B) Change in tumor volume from baseline to day 7 across cohorts.** 300 µg of activated MENPs demonstrated significant decrease in tumor volumes as compared to both non-activated MENPs and sham controls (One-way ANOVA, F = 5.731, df = 3, *P* = 0.007). **(C) Change in T_2_^*^ relaxation times from baseline to day 7 across cohorts.** No significant differences were observed across cohorts (Kruskal-Wallis test, H = 4.407, df = 2, *P* = 0.110). However, since the pilot study demonstrated significant outcomes, exploratory post-hoc pairwise comparisons were performed and demonstrated a significant decrease in the 600 µg MENP cohort as compared to the sham controls. Significant associations are indicated as follows: * for *P* < 0.05, ** for *P* < 0.01, and *** for *P* < 0.001. Blue asterisks indicate unadjusted (Dunn’s & LSD) significance, and green asterisks indicate adjusted (Bonferroni’s) significance. Abbreviations: ANOVA: analysis of variance; KW: Kruskal-Wallis; MENP: magnetoelectric nanoparticle.


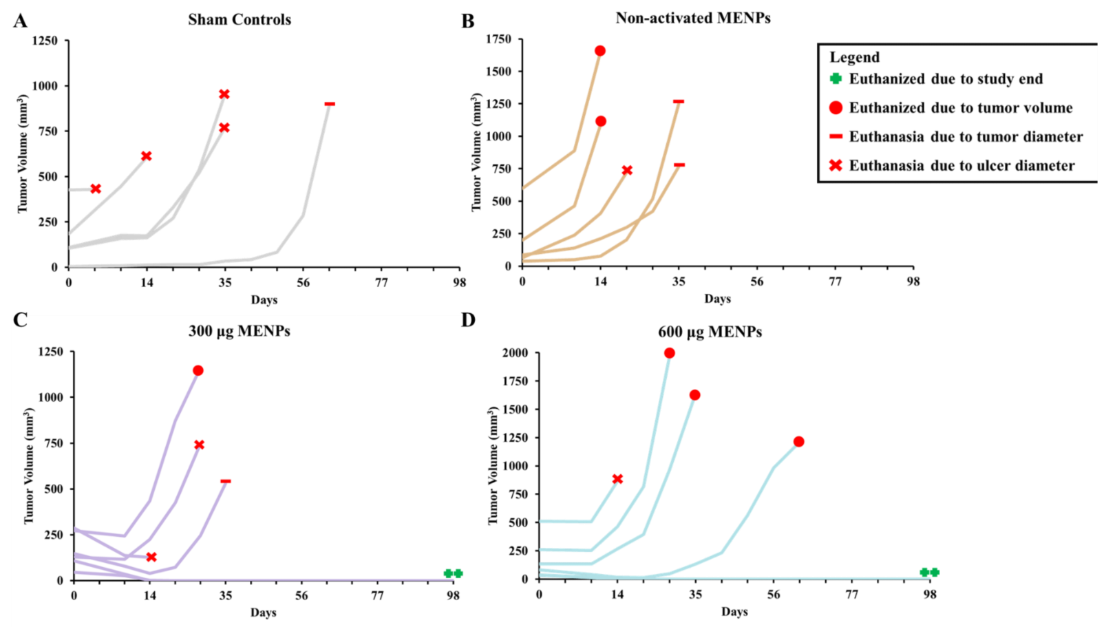


**Fig. S8: Longitudinal progress of individual mouse tumor volumes.** **(A-B) Tumor volume changes in the sham and non-activated MENP control cohorts.** Tumor volumes generally increased over time, reflecting uninhibited tumor growth in the absence of treatment. **(C-D) Tumor volume changes in the 300 µg and 600 µg MENP cohorts.** All mice demonstrated either tumor shrinkage or stabilization early in the study. In each cohort, 2 mice (33%) achieved an early complete response that remained stable throughout the study duration. However, tumors that did not achieve a complete response eventually resumed growth, ultimately reaching a euthanasia endpoint. Abbreviation: MENP: magnetoelectric nanoparticle.


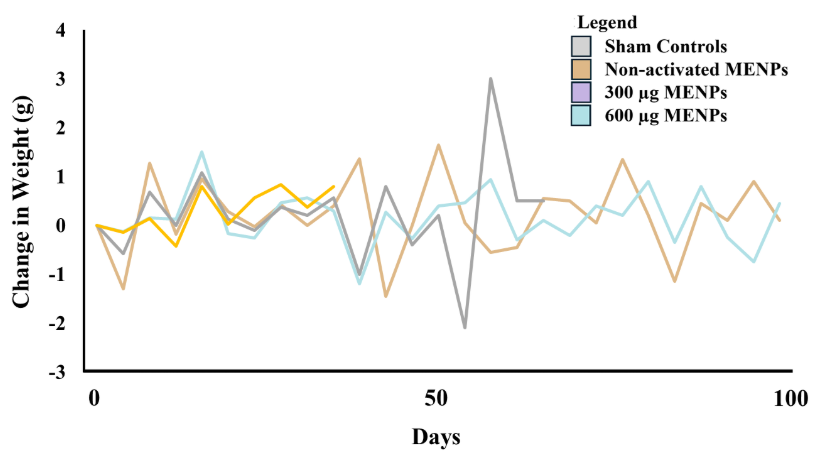


**Fig. S9: Change in weight over time across cohorts.** The figure shows the mean change in weight (g) for mice in each cohort (Sham Controls, Non-activated MENPs, 300 μg MENPs, and 600 μg MENPs) measured at semi-weekly intervals throughout the study. Statistical analysis using one-way ANOVA revealed no significant differences in weight changes between any cohorts at any time point (*P* > 0.05 for all comparisons). Significant associations are indicated as follows: * for P < 0.05, ** for P < 0.01, and *** for P < 0.001.Abbreviations: MENP: magnetoelectric nanoparticles.

 
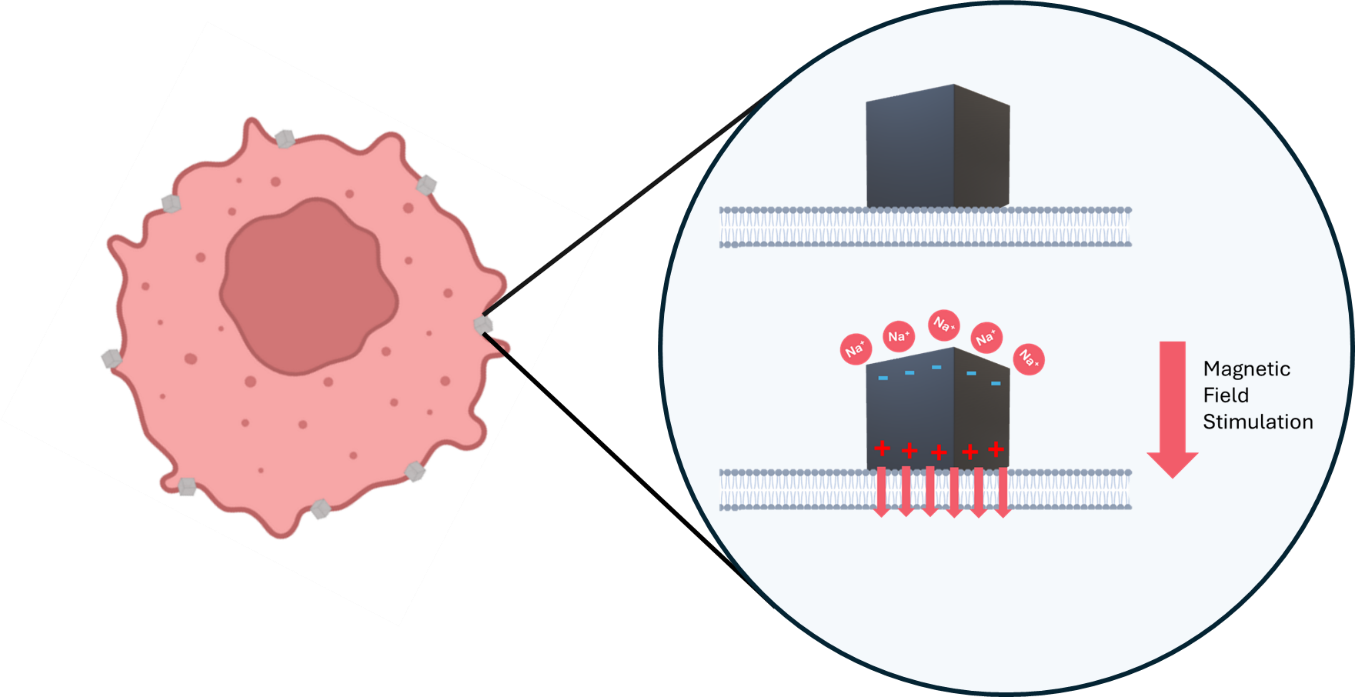


**Fig. S10: Diagramatic representation of MENP activation at the cell membrane.** The figure shows the generation of electric fields at the surface of the cell membrane. As the core puts strain on the shell, electrons and holes realign in the piezoelectric to generate net charges. The abundant ions in the microenvironment screen the field on one side of the particle, but where the MENP makes contact with the membrane, the charge isn’t screened, instead transferring charge to the membrane, acting like a capacitor discharging via one side.

.


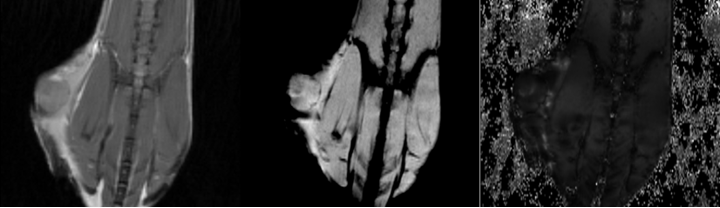


**Fig. S11: Differences in MRI sequences used for tumor imaging and analysis.** Representative images of murine flank tumors highlighting the visual differences among T_1_-weighted (left), T_2_^*^-weighted (middle), and T_2_^*^ mapping (right) sequences. While the T_1_-weighted and T_2_^*^-weighted sequences provided superior anatomical visualization of the tumor and surrounding tissues, the T_2_^*^ mapping sequence lacked clear anatomical details but was essential for quantitative measurement of intratumoral relaxation times.


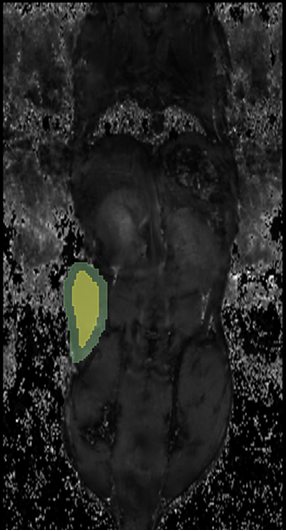


**Fig. S12: Contouring example**. Coronal slice from a baseline (day 0) T_2_^*^ map. Green segment represents volumetric contour, and yellow segment represents a isotropic 1 mm contraction to control for subtle motion and volumetric averaging artifact. Although this strategy was necessary to reduce artifact, it may underestimate true impact of MENPs on relaxation times as they tended to be located on the lateral periphery of the tumor (i.e., within 1 mm subtraction region) where the strongest magnetic fields of the targeting magnet was located.


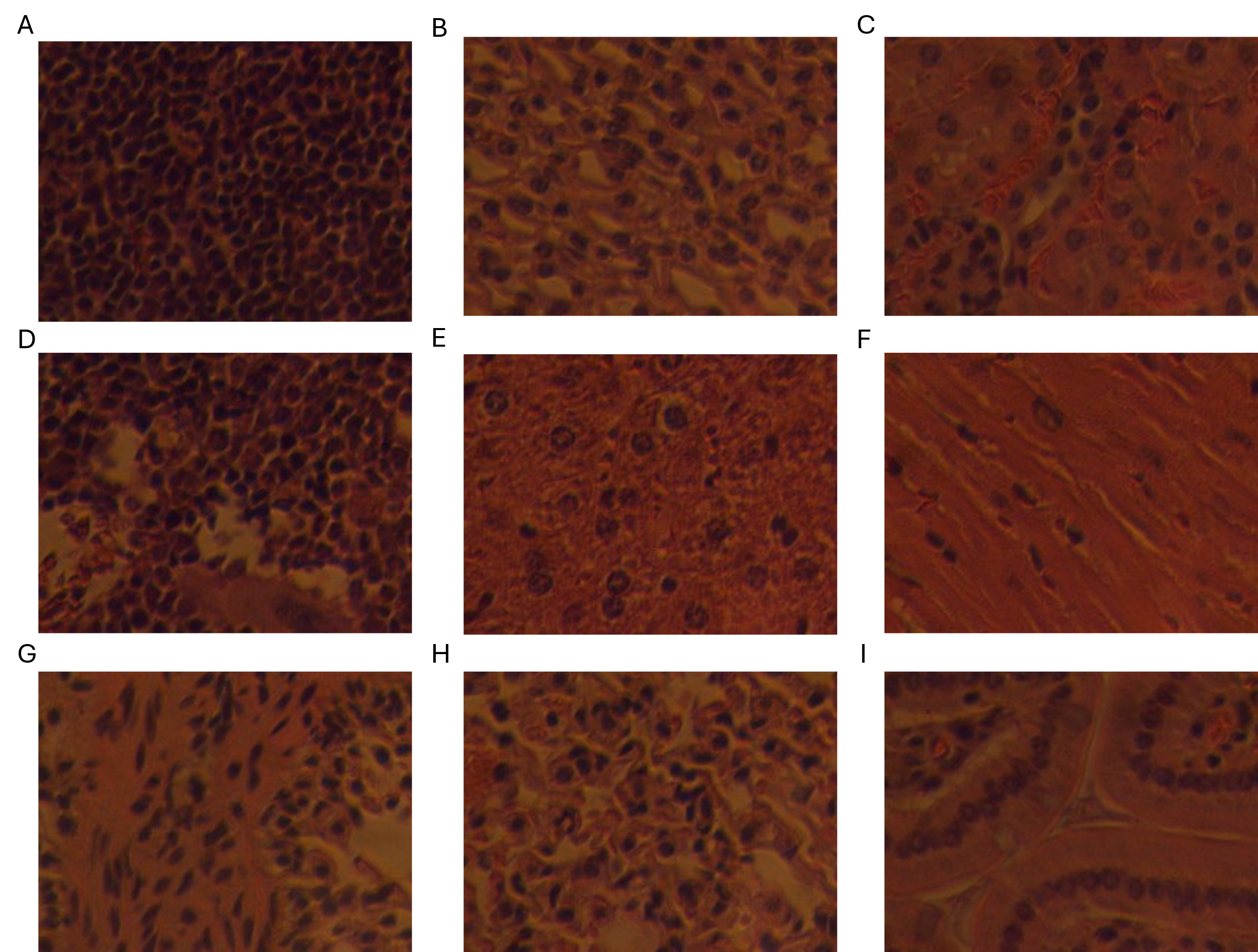
**Fig. S13: HE Stained Histology Images** A. Spleen B. Right Kidney C. Left Kidney D. Primary Tumor E. Liver F. Heart G. Right Lung H. Left Lung I. Bowel

**Table S1. Pilot study median tumor volume, relaxometry, and weight data.**

| **Metric** | **60 µg MENPs**   n/median (IQR) | **300 µg MENPs**   n/median (IQR) | **600 µg MENPs**   n/median (IQR**)** | **Sham Controls**   n/median (IQR) |
| --- | --- | --- | --- | --- |
| Cohort size, mice | 5 | 5 | 5 | 3 |
| Baseline tumor volume, mm³ | 87.7  (67.8-110.8) | 152.3  (119.5-155.8) | 53.4  (41.4-88.1) | 41.2  (35.9-69.2) |
| Day 7 tumor volume, mm³ | 97.9  (66.2-188.4) | 76.6  (60.0-126.7) | 26.6  (20.9-43.8) | 81.1  (68.9-171.0) |
| Baseline tumor T_2_^*^ relaxation time, ms | 12.4  (10.3-13.8) | 10.7  (8.9-12.8) | 10.3  (9.2-11.3) | 7.7  (7.0-9.1) |
| Activation MRI (Day 2) tumor T_2_^*^ relaxation time, ms | 12.0  (10.8-13.2) | 6.9  (5.2-7.5) | 6.1  (6.0-6.1) | 12.3  (10.9-12.7) |
| Baseline mice weights, g | 26.3  (22.9-26.5) | 25.3  (21.9-25.6) | 25.7  (21.9-26.1) | 25.2  (22.8-26.3) |
| Day 7 mouse weights, g | 24.7  (20.9-25.5) | 25.5  (21.7-25.5) | 24.7  (20.9-25.5) | 26.7  (23.7-26.9) |
| Abbreviations: IQR: interquartile range; MENP: magnetoelectric nanoparticle | | | | |

**Table S2. Confirmatory study tumor volume, relaxometry, and weight data.**

| **Metric** | **300 µg MENPs**   n/median (IQR) | **600 µg MENPs**   n/median (IQR**)** | **Non-activated MENPs**  n/median (IQR) | **Sham Controls**   n/median (IQR) |
| --- | --- | --- | --- | --- |
| n | 6 | 6 | 5 | 5 |
| Baseline tumor volume, mm³ | 137.1  (112.9-241.1) | 108.0  (49.2-227.9) | 84.9  (38.2-199.1) | 107.5  (80.3-128.0) |
| Day 7 tumor volume, mm³ | 97.5  (45.5-131.3) | 86.5  (23.3-223.4) | 140.3  (66.1-463.7) | 166.0  (119.8-242.8) |
| Baseline tumor T_2_^*^ relaxation time, ms | 11.2  (9.5-13.6) | 10.1  (8.9-14.4) | - | 12.8  (12.0-13.0) |
| Activation MRI (Day 2) tumor T_2_^*^ relaxation time, ms | 9.9  (9.4-11.6) | 8.0  (5.9-9.0) | - | 12.5  (10.2-14.3) |
| Baseline mice weights, g | 20.1  (19.9-21.5) | 20.4  (20.3-21.0) | 20.7  (20.6-20.7) | 20.7  (20.2-20.8) |
| Day 7 mouse weights, g | 20.3  (19.1-20.6) | 20.8  (20.5-20.9) | 19.9  (19.8-20.4) | 20.6  (19.9-20.9) |
| Abbreviations: IQR: interquartile range; MENP: magnetoelectric nanoparticle | | | | |

**Table S3. Confirmatory study survival data.**

| **Metric** | **300 µg MENPs**   n/median (IQR) | **60 µg MENPs**   n/median (IQR**)** | **Non-activated MENPs**  n/median (IQR) | **Sham Controls**   n/median (IQR) |
| --- | --- | --- | --- | --- |
| n | 6 | 6 | 5 | 5 |
| Survival time, days | 32.5  (29.0-83.8) | 42.8  (30.8-90.3) | 35.0  (15.0-35.0) | 35.0  (14.0-35.0) |
| Tumor volume endpoint reached^*^ | 1 | 3 | 2 | 0 |
| Tumor diameter endpoint reached^**^ | 1 | 0 | 2 | 1 |
| Ulcer diameter endpoint reached^***^ | 2 | 1 | 1 | 4 |
| Tumor complete response reached | 2 | 2 | 0 | 0 |
| Time to complete response, days | 19.0  (17.5-20.5) | 16.0  (16.0-16.0) | - | - |
| Abbreviations: IQR: interquartile range; MENP: magnetoelectric nanoparticle | | | | |
